# Differential associations between education and blood pressure by gender and race

**DOI:** 10.1186/s12889-025-23409-5

**Published:** 2025-07-02

**Authors:** Lucia Pacca, Amanda M Irish, Catherine dP Duarte, Alicia R Riley, Mark J Pletcher, Zinzi D Bailey, Anusha M Vable

**Affiliations:** 1 Department of Family and Community Medicine, University of California San Francisco, Pride Hall, 2540 23rd Street, 5th Floor, 94110 San Francisco, CA USA; 2 Department of Epidemiology and Population Health, Stanford University, Stanford, CA USA; 3 Sociology Department, University of California Santa Cruz, Santa Cruz, CA USA; 4 Department of Epidemiology & Biostatistics, University of California San Francisco, San Francisco, CA USA; 5 Department of Epidemiology and Community Health, University of Minnesota, Minneapolis, MN USA; 6 Philip R. Lee Institute for Health Policy Studies, University of California San Francisco, San Francisco, CA USA

**Keywords:** Cardiovascular disease, Blood pressure, Hypertension, Education, Differential returns, Racial and ethnic groups, Gender, Racial and gender inequalities

## Abstract

**Background:**

Previous research suggests education is inversely associated with blood pressure, but little work has examined whether this relationship differs by race and gender jointly. Identifying the most vulnerable groups may inform hypertension prevention strategies. In this population-based study, we investigate the association between education and blood pressure overall and across race-by-gender subgroups.

**Methods:**

Our analytic sample included participants aged 50 + to the US Health and Retirement Study data from 2006 to 2008 (*N* = 24,526). Our exposure was education, measured as self-reported years of schooling and modeled as a spline with a knot and discontinuity at 12 years representing high school diploma. We used generalized estimating equations to estimate the relationship between education and repeated measurements of two blood pressure outcomes: systolic blood pressure (SBP) and hypertension (HTN), then included race-by-gender interactions with education to evaluate differential associations. All models were adjusted for age, birthplace, parents’ education, and survey year.

**Results:**

Mean age was 64.4 years, mean SBP was 129.9 mmHg, and HTN prevalence was 63.1%. Overall, below 12 years, each additional year of education was not associated with blood pressure, while twelve years of schooling was associated with lower blood pressure (b=-1.02; 95% CI: -2.04, 0.00 for SBP) and each additional year of education after 12 years was associated with lower SBP and lower odds of HTN (e.g., SBP: b=-0.75 mmHg; 95% CI: -0.88, -0.62). We observed some differential relationships by demographic subgroup such that, among Black men, 12 years of education predicted higher odds of HTN compared to White men (interaction OR = 1.60; 95% CI: 1.02, 2.52), and each additional year of education after 12 years was associated with larger SBP benefits for White, Hispanic and Black women compared to White men.

**Conclusions:**

We found an overall protective relationship between more education and blood pressure/hypertension such that each additional year of college education was associated with lower blood pressure/hypertension, particularly among White and Hispanic women. However, we also found evidence of diminished benefits to high school degree attainment among Black men compared to other groups in hypertension prevalence.

**Supplementary Information:**

The online version contains supplementary material available at 10.1186/s12889-025-23409-5.

## Introduction

High blood pressure (hypertension) is a leading cause of cardiovascular disease (CVD) and cardiovascular mortality. In the US, nearly half of the adult population (47%, 116 million) has hypertension, with greater prevalence among older adults [1]. The prevalence of hypertension is increasing across populations, with notable inequities [2, 3]. Black adults are burdened with a significantly higher hypertension prevalence (59%) than White (47%) and Hispanic adults (44%) [4]. Previous research also showed gender differences in the incidence and severity of hypertension such that men tend to have a higher incidence of hypertension and worse blood pressure control than women of the same age [5, 6]. Identifying upstream risk factors for hypertension overall as well as racial and gender inequities may help illuminate the underlying mechanisms and inform prevention strategies.

**Fig. 1:**
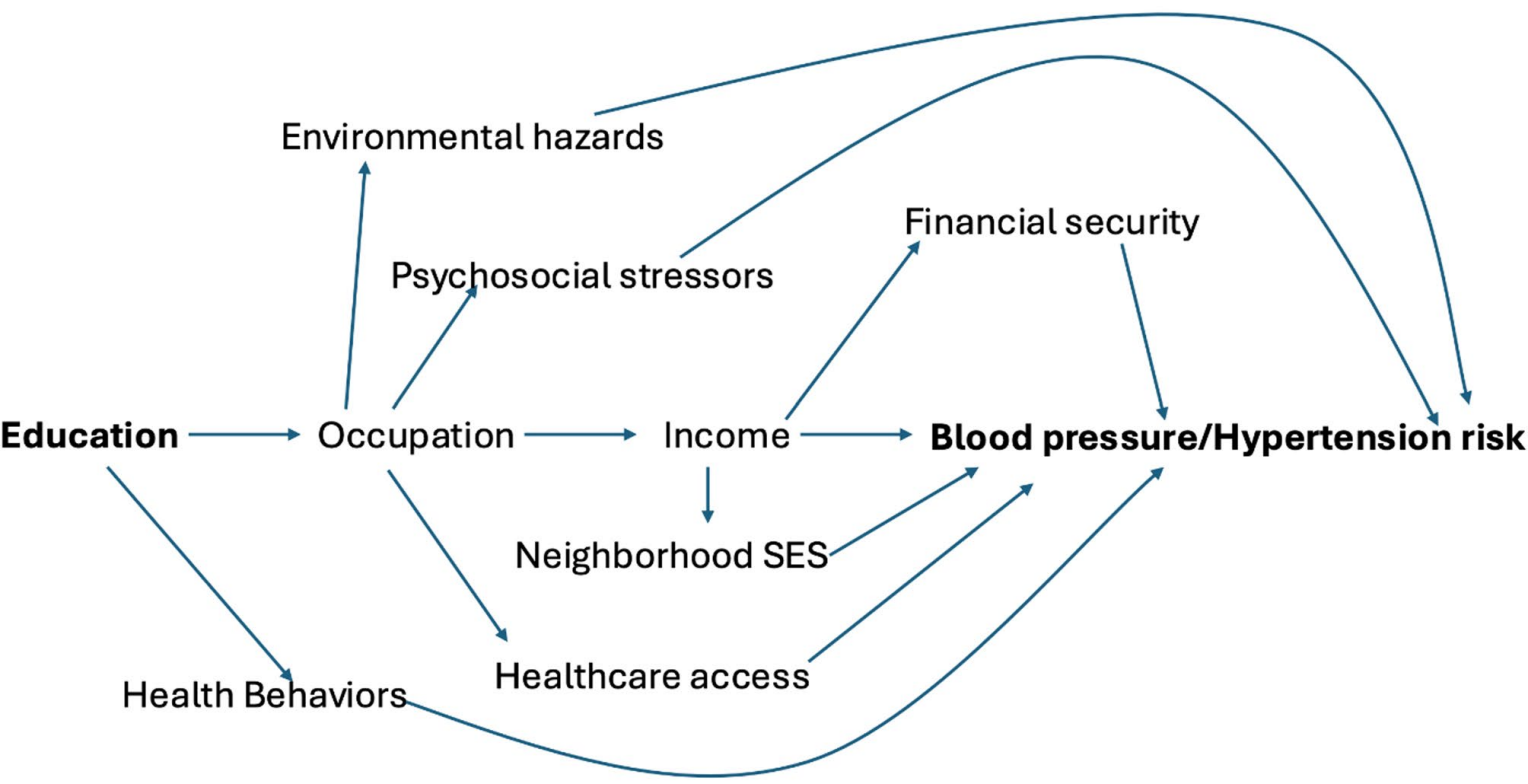
Main hypothesized mechanisms linking education to hypertension/blood pressure. *Legend*: This Figure visualizes the main hypothesized mechanisms linking education with later-life blood pressure/hypertension risk. Education is a predictor of occupation; work environments determine exposure to psychosocial stressors and environmental hazards [11]; income; healthcare access and quality (through employer-sponsored health coverage). Education is also related to health behaviors; for example, knowledge and cognitive ability are inversely related to harmful behaviors such as smoking. Income, achieved through occupation, influences where people choose to live (e.g., neighborhood socio-economic status), which can determine access to resources and social networks. Higher income can reduce financial insecurity, with lower subsequent stress, a determinant of hypertension

Educational attainment is among the upstream factors most strongly and consistently associated with hypertension and CVD risk, with higher educational attainment predicting a lower cardiovascular risk [7]. Years of schooling and degree attainment have been associated with lower blood pressure, even after adjusting for early life characteristics such as childhood health and childhood socioeconomic status (SES) [7–10]. The main hypothesized mechanisms linking higher education with lower hypertension risk are showed in Fig. 1. These include subsequent access to work environments with more autonomy, fewer psychosocial stressors and environmental hazards [11]; higher income [12, 13]; better healthcare access and quality; more physical activity and healthy food access; homeownership in better resourced communities resulting in stronger social cohesion [14, 15]. However, the cardiovascular benefits of educational attainment may not be equally shared across gender and racial subgroups given the omnipresence of sexism and racism at various levels, including structural [16].

Experiences of racial discrimination extend to various settings, such as employment. An existing body of research demonstrates how racial discrimination in hiring and retention disproportionately targets Black men, resulting in higher rates of unemployment, lower wages, and fewer promotion opportunities compared to White men with similar levels of education [17, 18]. Perceived workplace discrimination has also been linked to psychological stress, which may increase the risk of hypertension [19, 20] and poorer health management. In addition, improved access to healthcare, facilitated by health insurance coverage, is generally linked to a lower likelihood of uncontrolled hypertension. However, access to healthcare is not uniformly health benefiting as patients may experience discrimination at the health system level, the burden of which has been shown to be highest among Black Americans and other minoritized groups and is linked to lower quality of care, under-treatment, and reduced access and adherence to medications [21]. 

To date, most studies investigating differential relationships between education and blood pressure have considered either race and ethnicity or gender separately. This work found that educational attainment and educational quality are more strongly protective of hypertension among women as compared to men [21, 22]attributing these findings to individual-level mechanisms such as more frequent physician visits and greater lifestyle modifications [23]. Such evidence is in line with the Resource Substitution Theory [24]which posits that education improves health more for structurally minoritized groups (in particular women) relative to more advantaged groups (i.e., White men) because the latter have greater access to health-promoting alternatives to education, such as power, authority, and earnings. This, in turn, increases the relative importance of the resources that minoritized groups have access to, such as education, and makes them more critical for their health.

In contrast, several studies have found that the association between higher education and lower hypertension found among non-Hispanic White adults in the U.S. is not present for racially- and ethnically-minoritized groups, particularly for Black Americans [25–27]. This finding is instead consistent with the theory of Minorities’ Diminished Returns [28–30]which posits that education and other SES indicators are less protective of health for racially- and ethnically-minoritized adults relative to non-Hispanic White adults due to exclusionary processes such as: residential segregation [31]; structural divestment from schools serving predominantly students of color (e.g., fewer financial resources and learning opportunities); reduced access to healthcare and medical undertreatment [32]; and increased exposure to discrimination and stress in less diverse educational and workplace environments [30, 33]. Taken together, prior studies find inconsistent patterns in the educational attainment-hypertension association across experiences of structural marginalization shaped by gender compared to those shaped by race. Further exploration of these patterns is warranted [34].

Few studies have tested for differential education-hypertension effects at the intersections of race/ethnicity and gender. Intersectionality theory - rooted in Black feminist thought - posits that interlocking systems of power and oppression (e.g., racism, sexism and classism) collectively structure the environments with which individuals and the resources to which they have access [35–37]. In doing so, these interlocking systems produce corresponding patterns of health and well-being observed at the intersections of individual social identities (e.g., race, ethnicity and gender). Far from representing the simple addition of social identities such as race and gender, the intersectionality perspective asserts that race and gender constitute each other such that one identity alone (e.g., gender) cannot explain the unique experience created at the intersection with the other identities in the face of social discrimination [35, 38, 39]. The interlocking systems of racism and sexism may operate jointly to alter observed returns to educational attainment for multiply marginalized people, with implications for later-life health [35]. For example, Black women’s experiences of these interlocking systems may be distinct from – and not simply summative of – that of White women or Black men. From differential experiences within their schools, to subsequent access to jobs, adequate income, or healthcare, the distributions of these health-relevant experiences among older adults in America cannot be understood without an intersectional lens [40]. Further, these stressors, and their material and psychosocial consequences, may persist, even as educational status increases, contributing to racial and gender inequities in chronic health conditions. In public health research, interaction terms and stratified analyses have been the primary quantitative techniques used to study intersectional inequities [41]. Such approaches permit exploration of the compounding nature of these interlocking systems of oppression (e.g., racism and sexism) as embodied “at the microlevel of individual experience” (e.g., race and gender) (cite). Applying this intersectionality framework to social determinants of health research allows us to better document the nuanced ways in which health inequities present across social groups, an important first step towards addressing their causes.

Motivated by this intersectionality framework and using data from the Health and Retirement Study, our aim was to understand whether educational attainment’s associations with blood pressure and hypertension differ at the intersections of gender and race/ethnicity. We further aimed to identify subgroups for whom adverse effects may be most pronounced to inform research and policy avenues for reducing cardiovascular health inequities.

## Methods

### Study population

Data came from the US Health and Retirement Study (HRS). The HRS is a nationally representative, longitudinal study collecting biennial surveys of US adults aged 50 + years and their spouses. In addition to the nationally representative, multi-stage area probability sample (the core sample), the HRS was designed to oversample Black and Hispanic populations as well as Florida residents.

In 2006, the HRS initiated an Enhanced Face-to-Face (EFTF) interview that consisted of a set of physical performance tests, including blood pressure measurements. Half of the 2006 sample was randomly selected for the EFTF interview, and the other half followed in 2008. Physical measures were subsequently collected every 4 years for respondents. Among participants aged 50 + years in 2018 and eligible for physical measurements at one or more time points (*n* = 28,348), we excluded individuals with no available BP measurements between 2006 and 2018 (*n* = 3,580) and those with missing covariates (*n* = 242). Our final analytic sample included 24,526 h participants (86.5% of eligible participants), comprising 51,692 observations (2.1 mean observations per person).

### Exposure: education

We used self-reported years of schooling as a measure of education (0–17+). In our analytic sample, *N* = 688 respondents with fewer than five years of schooling were recoded to 5 years due to data sparseness. Unstable estimates, especially at the tails of the distribution, can result in biased estimates for linear exposures. We operationalized education as a linear spline with a knot and discontinuity at 12 years, which typically coincides with high school degree attainment, a credential previously found to predict several health outcomes (the coding of the spline is illustrated in Table S1 in the supplemental material) [42]. This operationalization is meaningful for considering policy implications as estimates for those with fewer than 12 years reflects K-12 schooling, estimates at 12 years reflects a high school diploma, and estimates for more than 12 years of education reflects post-secondary (e.g. college) schooling [43]. As suggested by previous research, using a spline allows retaining the continuity of the education variable, testing whether each additional year of education is differentially associated with BP, while at the same time assessing whether the high school diploma represents a threshold associated with a different BP outcome [44, 45]. In addition to this theoretical justification for a knot at 12 years, we found that this operationalization also resulted in better model fit than other functional forms, based on the QIC criterion shown in the Supplement (Figure S1, Table S2).

### Outcomes

Our outcomes included repeated measurements of systolic blood pressure (SBP) and hypertension (HTN). SBP was chosen over diastolic blood pressure because extensive research has shown that it is more strongly associated with cardiovascular risk [46]. The HRS interviewers used Omron HEM-780 N monitors to measure systolic blood pressure readings from participants while they were seated with both feet on the floor. Before taking the blood pressure measurements, interviewers were given detailed instructions on removing participants’ bulky clothing, proper sitting position for blood pressure measurement, use of the measuring device and reading and recording of systolic and diastolic blood pressure [47]. Blood pressure readings were taken three times between 45 and 60 s apart from a participant’s supported left arm with the palm facing upward. As recommended by the American Heart Association, if there were three non-missing SBP measurements, we used the mean of the second and third measurements [48]. If there were only one or two non-missing SBP measurements, we calculated the mean of all available measurements.

Hypertension was operationalized as a dichotomous variable. Based on the Seventh Report of the Joint National Committee on Prevention, Detection, Evaluation, and Treatment of High Blood Pressure [49], participants were classified as hypertensive if they had systolic blood pressure of at least 140 mmHg or diastolic blood pressure of at least 90 mmHg. Participants who self-reported taking anti-hypertensive medication were classified as having hypertension even when their blood pressure was lower than 140/90 mmHg.

### Effect modifiers

We evaluated effect modification by race/ethnicity and self-reported gender. Gender was operationalized as a binary indicator, given the responses recorded in the HRS. Participants self-identified their race as “White”, “Black/African American,” or a range of “other” races, including American Indian, Alaska Native, Asian, Native Hawaiian, and Pacific Islander, which were collapsed in the public dataset to protect participant confidentiality due to small sample sizes. Only Hispanic/Latino ethnicity was assessed; participants self‐identified their ethnicity as “Yes,” “No,” or “Don’t know” in response to the question “Do you consider yourself Hispanic or Latino?“. Participants’ responses to the race and ethnicity items were combined into 4 race/ethnicity categories: non‐Hispanic Black, non‐Hispanic White, Hispanic and non-Hispanic other race/ethnicity. The “other” race/ethnicity category was included in analysis, but results were not presented due to ambiguous interpretation. Race and ethnicity were used as a proxy for exposure to social stratification systemic racism, which causes unequal distribution of resources and adverse health outcomes among racialized persons [50]. We modeled race/ethnicity-by-gender interactions using interaction terms between race/ethnicity and gender. We evaluated differential associations between education and SPB/HTN by interacting each race by gender group with the three components of the education spline (< 12 years of education, >=12 years of education (yes/no), > 12 years of education). We set White men, the most socially advantaged group, as the reference group for our analyses to highlight differential associations more clearly with education among structurally minoritized groups.

### Covariates

We accounted for age (in years) [51, 52], place of birth (foreign birth, southern US, non-southern US, or not specified), year of blood pressure measurement (2006–2018), and mother’s and father’s education [53]. Mother’s and father’s education were operationalized as years of schooling, and missing values (9.6% for mother’s education and 17.0% for father’s education) were replaced with the sample average. We included missing indicators for mother’s and father’s education as additional confounders. As theorized and applied in previous work using HRS data, we used the missing indicator method to impute mother’s and father’s education, since missing parental education may indicate lower childhood social capital (e.g., not having a parent present in the household) [54–57].

In SBP analyses, we did not adjust for anti-hypertensive medications because medication usage is a potential mediator of the relationship between education and blood pressure.

### Statistical analyses

We used generalized estimating equations (GEE) with an exchangeable correlation structure to evaluate the association between education and repeated measures of SBP and hypertension. We specified the identity link for SBP because it is normally distributed, and the logit link for hypertension because it is a binary variable. The base model was adjusted for race, gender, and all confounders. In a separate model, we included interactions between education and race/ethnicity by gender groups, using White men as the reference group. The constant and main effects represent the reference group (White men), while the interaction terms represent the difference in the association between education and blood pressure for each group compared to White men. In addition, we evaluated the joint significance of all the included interaction terms using a global f-test. We also conducted analyses stratified by race/ethnicity-by-gender groups. Interaction and stratified analyses were adjusted for all the confounders listed above.

### Robustness checks

We conducted several sensitivity analyses. First, we conducted two robustness checks on the outcome using different operationalizations: [1] due to changes in hypertension guidelines set by the American College of Cardiology/American Heart Association (ACC/AHA) before the end of the study period, we used 130/80 mmHg as an alternative threshold to determine hypertension [58]; [2] we used diastolic blood pressure as the outcome. [3] As a robustness check on the exposure, we estimated the relationship between education and cardiovascular risk using self-reported degree attained (less than high school/GED, high school, college or higher) rather than continuous years of schooling. Finally, we conducted two robustness checks on the analytic sample: [4] we conducted multiple imputations by chained equations (MICE) with 30 iterations on missing outcomes, exposures and covariates to minimize potential selection bias. This approach relies on the assumption that the variables are missing at random and leverages existing relationships between variables in the observed data to impute the missing data [59]. [5] We conducted a sensitivity analysis excluding non-US born participants (*N* = 3,450), who are more likely to have received their education, or a portion of it, in their home countries. Education achieved abroad may not be equivalent to education achieved in the United States; for example, 12 years of education do not correspond to a high school diploma in all the world countries [60].

All the analyses were conducted using STATA version 16 [61] and all code was reviewed by an independent coder (the second author) as is recommended practice [62].

## Results

Baseline characteristics of our analytic sample are presented in Table 1. Participants’ mean age was 64.4 (sd = 10.6) years, and 57% of participants were women. On average, participants had completed 12.7 (sd = 2.9) years of schooling. The analytic sample was 63.1% White, 19.4% Black, 13.6% Hispanic and 3.9% of other race/ethnicity (including American Indian, Alaskan Native, Asian, Pacific Islander and not specified). Mean SBP at baseline was 129.9 mmHg (sd = 20.6), and 63.1% of participants had hypertension.

**Table 1:** Baseline sample characteristics, overall and by race-ethnicity and gender sub-groups

	Overall	White Men	White Women	Black Men	Black Women	Hispanic Men	Hispanic Women
	*N* = 24,526	*N* = 6797	*N* = 8687	*N* = 1873	*N* = 2897	*N* = 1439	*N* = 1888
Age, mean (sd)	64.4 (10.6)	66.3 (10.7)	66.4 (11.1)	61.3 (8.9)	61.7 (9.5)	60.9 (8.8)	60.8 (9.1)
School years, mean (sd)	12.7 (2.9)	13.5 (2.7)	13.2 (2.4)	12.3 (2.8)	12.6 (2.6)	10.4 (3.8)	10.3 (3.8)
Birth place							
US not south, N (%)	12,288 (50.1%)	4552 (67.0%)	5689 (65.5%)	450 (24.0%)	684 (23.6%)	265 (18.4%)	355 (18.8%)
Southern birth, N (%)	8227 (33.5%)	1888 (27.8%)	2528 (29.1%)	1166 (62.3%)	1841 (63.5%)	254 (17.7%)	311 (16.5%)
Immigrant, N (%)	3450 (14.1%)	285 (4.2%)	381 (4.4%)	149 (8.0%)	175 (6.0%)	898 (62.4%)	1179 (62.4%)
US not specified, N (%)	561 (2.3%)	72 (1.1%)	89 (1.0%)	108 (5.8%)	197 (6.8%)	22 (1.5%)	43 (2.3%)
Mother’s education, mean (sd)	9.9 (3.7)	10.8 (3.0)	10.6 (3.1)	9.9 (3.3)	9.6 (3.3)	6.3 (4.5)	6.2 (4.5)
Father’s education, mean (sd)	9.6 (3.8)	10.4 (3.5)	10.2 (3.5)	9.1 (3.4)	8.9 (3.2)	6.7 (4.5)	6.9 (4.6)
Missing mother’s education							
Yes	22,174 (90.4%)	6245 (91.9%)	8126 (93.5%)	1604 (85.6%)	2478 (85.5%)	1240 (86.2%)	1640 (86.9%)
No	2352 (9.6%)	552 (8.1%)	561 (6.5%)	269 (14.4%)	419 (14.5%)	199 (13.8%)	248 (13.1%)
Missing father’s education							
Yes	20,349 (83.0%)	6047 (89.0%)	7673 (88.3%)	1333 (71.2%)	1972 (68.1%)	1133 (78.7%)	1426 (75.5%)
No	4177 (17.0%)	750 (11.0%)	1014 (11.7%)	540 (28.8%)	925 (31.9%)	306 (21.3%)	462 (24.5%)
Systolic blood pressure, mean (sd)	129.9 (20.6)	132.0 (18.8)	126.6 (20.5)	136.3 (20.7)	131.9 (23.2)	133.7 (19.8)	126.3 (20.6)
Diastolic blood pressure, mean (sd)	80.2 (11.9)	79.6 (11.4)	78.9 (11.5)	83.7 (12.9)	82.8 (13.1)	81.4 (11.8)	79.1 (11.4)
Hypertension							
Yes, N (%)	9051 (36.9%)	2513 (37.0%)	3614 (41.6%)	487 (26.0%)	660 (22.8%)	555 (38.6%)	807 (42.7%)
No, N (%)	15,475 (63.1%)	4284 (63.0%)	5073 (58.4%)	1386 (74.0%)	2237 (77.2%)	884 (61.4%)	1081 (57.3%)

Summary statistics by race/ethnicity and gender showed differences in key characteristics. On average, White women had the highest baseline age (66.4, sd = 11.1), and Hispanic women the lowest (60.8, sd = 9.1). White men had the highest mean years of schooling (13.5, sd = 2.7) and Hispanic women the lowest (10.3, sd = 3.8). Hypertension prevalence was highest among Black women (77.2%) and lowest among Hispanic women (57.3%).

### Base model results

In the base model (Table 2), each additional year of education up to 12 years was not associated with blood pressure. The spline knot at 12 years of education, indicating high school diploma, was associated with lower SBP (β=-1.02, 95% CI= -2.04, 0.00) and lower odds of hypertension (OR = 0.92, 95% CI = 0.81, 1.03). This indicates that, compared to not completing high school (being just below 12 years of education), completing high school (achieving 12 years of education) was associated with 1.02 mmHg lower SBP and 8% lower odds of having HTN; that is, the estimate from the knot in the spline can be interpreted as a “downward jump” in SBP and hypertension risk in correspondence to the high school credential. Each additional year of education after 12 years was associated, on average, with 0.75 mmHg lower SBP (95% CI= -0.88, -0.62) and with lower odds of hypertension (OR = 0.92, 95% CI = 0.91,0.94).

**Table 2:** Generalized estimating equation, base models

	Systolic Blood Pressure			Hypertension		
VARIABLES	β	95% CI	p	Odds ratio	95% CI	p
Education, per year < 12	-0.08	-0.33, 0.16	0.51	1.00	0.97, 1.03	0.93
Education > = 12 years (Y/N)	-1.02	-2.04, 0.00	0.05	0.92	0.81, 1.03	0.15
Education, per year > 12	-0.75***	-0.88, -0.62	0.00	0.93***	0.91, 0.94	0.00
Race and Ethnicity (Ref = White)						
Black	5.58***	4.93, 6.23	0.00	2.37***	2.19, 2.56	0.00
Hispanic	1.63***	0.77, 2.49	0.00	1.20***	1.08, 1.32	0.00
Gender (Ref = Male)						
Female	-4.82***	-5.25, -4.40	0.00	0.85***	0.80, 0.89	0.00
Father’s education	-0.08*	-0.15, -0.00	0.04	0.99***	0.98, 0.99	0.00
Mother’s education	0.04	-0.05, 0.12	0.37	1.00	0.99, 1.01	0.86
Missing mother’s education	0.21	-0.69, 1.12	0.65	0.95	0.86, 1.05	0.35
Missing father’s education	0.10	-0.59, 0.79	0.78	1.12**	1.04, 1.22	0.00
Birthplace (Ref = Non-Southern US)						
Southern birth	0.97***	0.47, 1.48	0.00	1.19***	1.12, 1.26	0.00
Immigrant	-0.16	-0.99, 0.67	0.71	0.81***	0.74, 0.89	0.00
US not specified	0.52	-0.97, 2.00	0.50	1.15	0.96, 1.37	0.13
Age	0.31***	0.29, 0.34	0.00	1.05***	1.04, 1.05	0.00
Year (Ref = 2006)						
2008	-0.02	-0.66, 0.63	0.96	1.01	0.95, 1.08	0.74
2010	0.51	-0.01, 1.02	0.05	1.12***	1.06, 1.17	0.00
2012	-1.90***	-2.52, -1.28	0.00	1.03	0.97, 1.10	0.36
2014	-2.37***	-2.92, -1.82	0.00	1.02	0.97, 1.08	0.38
2016	-3.00***	-3.62, -2.38	0.00	0.94	0.88, 1.00	0.07
2018	-3.79***	-4.38, -3.20	0.00	0.96	0.91, 1.01	0.14
Constant	113.43***	111.37, 115.50	0.00	0.12***	0.09, 0.15	0.00
Observations (Number of id)	51,693 (24,526)			51,693 (24,526)		

Education is operationalized as a spline using self-reported years of schooling (5–17). Mother’s and father’s education are parents’ years of schooling, and missing mother’s and father’s education are indicator variables. Statistical significance is indicated as following: *** P-value < 0.001, ** P-value < 0.01, * P-value < 0.05

### Interaction and stratified results

**Fig. 2:**
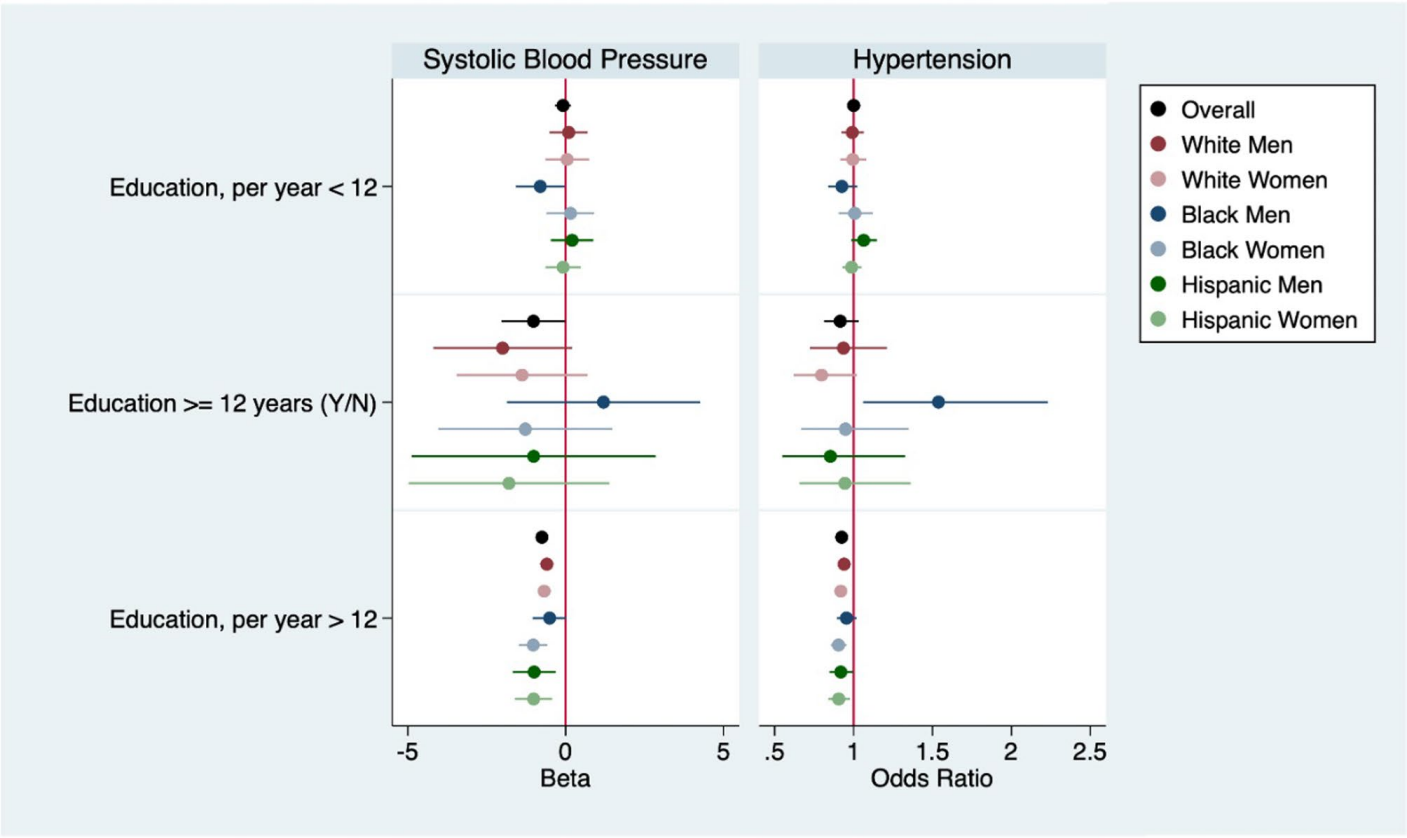
Generalized Estimating Equations, Stratified Results. *Legend*: Regressions are adjusted for age, birth place, mother’s and father’s education, missing indicators for mother’s and father’s education and year of outcome measurement. Education is operationalized as a spline using self-reported years of schooling (5–17). Coefficient plots indicate beta coefficients for systolic blood pressure and odds ratios for hypertension

**Fig. 3:**
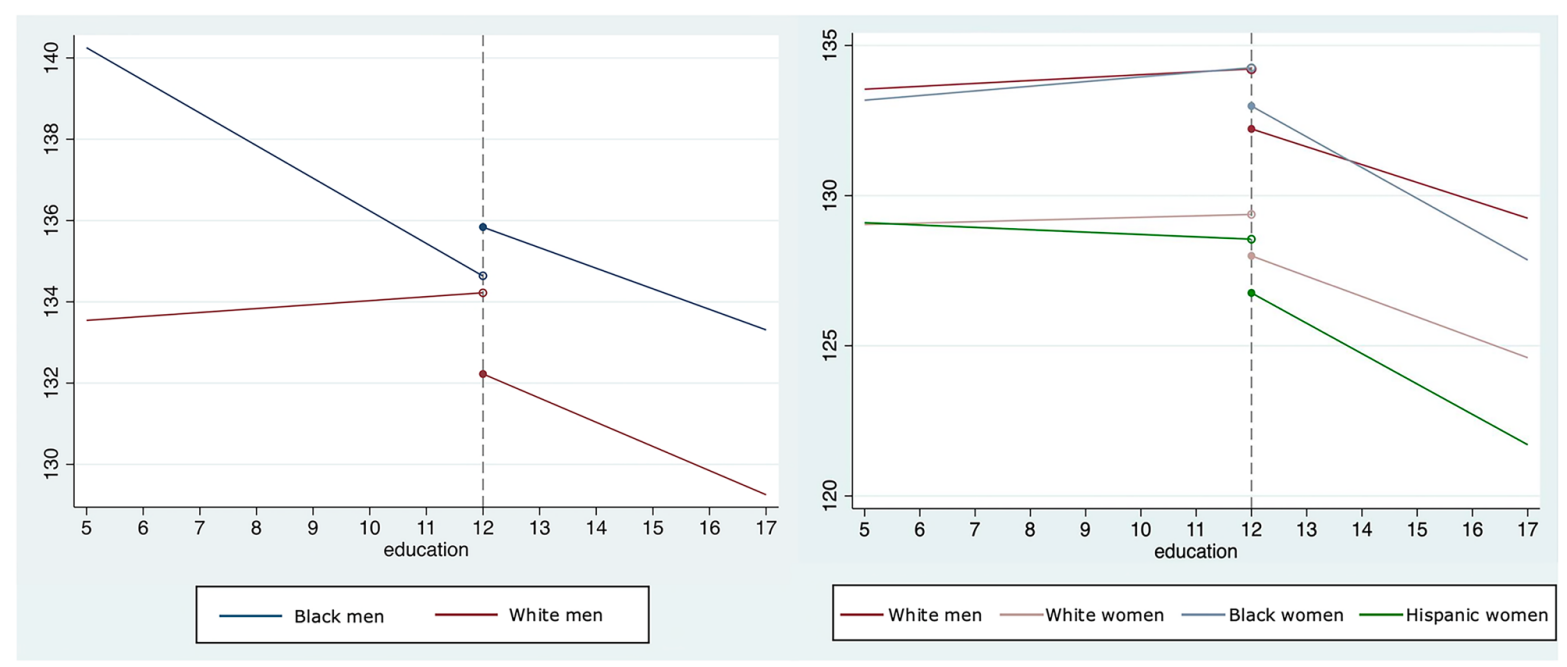
Adjusted means from stratified model estimating the association between education and SBP. *Legend*: Each line represents predicted adjusted means from generalized estimating equations estimating the association between education (operationalized as a spline) and SBP, stratified by race/ethnicity by gender group. The education spline is operationalized as the combination of three different components: education < 12 years (continuous years of schooling from 5 to right before 12), education > = 12 years (categorical variable, equal to 0 if years of schooling < 12, 1 otherwise), and education > 12 years (continuous years of schooling from 12–17). The two slopes indicate the association between each additional year of education (before and after achieving a high school diploma) and SBP, while the discontinuity indicates the association between achieving a high school diploma and SBP. The left panel shows results for Black men and White men to highlight the difference in the association between the high school credential and SBP (knot at 12 years of education). The right panel shows results for White men, White women, Hispanic Women and Black women to highlight differences in slopes for the association between education > 12 years and SBP

Results from interaction analyses are presented in Table 3 and visualized in Figures S2 and S3 (Supplemental material), while coefficients from stratified analyses are visualized in Figs. 2 and 3. There was little evidence of a differential relationship between education and race-by-gender among those with fewer than 12 years of schooling. Stratified results showed that achieving 12 years of education was associated with lower blood pressure and lower odds of hypertension, although the confidence intervals included the null, among each race by gender group except for Black men, for whom 12 years of education were associated with higher odds of hypertension. Results from interaction analyses indicated that, compared to White men, 12 years of education was associated with 60% higher odds of having hypertension among Black men (interaction OR = 1.60, 95% CI = 1.02, 2.52).

**Table 3:** Generalized estimating equation, interaction models

	Systolic Blood Pressure				Hypertension		
VARIABLES	β	95% CI	P-value	Odds Ratio	95% CI	P-value	
White women	-4.17**	-7.09, -1.25	0.01	1.01	0.72, 1.43	0.95	
Black men	2.99	-0.53, 6.50	0.10	1.27	0.84, 1.93	0.25	
Black women	2.18	-1.14, 5.50	0.20	2.46***	1.64, 3.70	0.00	
Hispanic men	3.18	-0.94, 7.29	0.13	1.49	0.93, 2.38	0.09	
Hispanic women	-2.73	-6.35, 0.88	0.14	1.05	0.70, 1.57	0.82	
Education, per year < 12 (White men)	0.15	-0.46, 0.76	0.63	0.99	0.92, 1.07	0.85	
White women*education, per year < 12	-0.25	-1.18, 0.67	0.59	0.99	0.89, 1.11	0.90	
Black men* education, per year < 12	-0.36	-1.33, 0.62	0.47	0.96	0.85, 1.08	0.46	
Black women* education, per year < 12	0.08	-0.89, 1.04	0.88	1.02	0.90, 1.16	0.71	
Hispanic men* education, per year < 12	-0.03	-0.92, 0.86	0.95	1.07	0.96, 1.18	0.21	
Hispanic women* education, per year < 12	-0.28	-1.10, 0.53	0.50	0.98	0.90, 1.08	0.74	
Education > = 12 years (Y/N, White men)	-1.44	-3.65, 0.78	0.20	0.97	0.75, 1.27	0.85	
White women*education > = 12 years	-0.00	-3.03, 3.03	1.00	0.82	0.57, 1.17	0.27	
Black men*education > = 12 years	2.50	-1.29, 6.28	0.20	1.60*	1.02, 2.52	0.04	
Black women*education > = 12 years	-0.19	-3.75, 3.36	0.91	0.95	0.61, 1.47	0.82	
Hispanic men* education > = 12 years	0.79	-3.68, 5.25	0.73	0.89	0.54, 1.49	0.67	
Hispanic women* education > = 12 years	-0.92	-4.83, 2.99	0.64	0.94	0.60, 1.46	0.78	
Education, per year > 12 (White men)	-0.54***	-0.74, -0.33	0.00	0.94***	0.92, 0.97	0.00	
White women*education, per year > 12	-0.33*	-0.61, -0.06	0.02	0.96*	0.93, 1.00	0.04	
Black men* education, per year > 12	0.23	-0.35, 0.80	0.44	1.03	0.96, 1.10	0.45	
Black women* education, per year > 12	-0.52*	-1.00, -0.04	0.03	0.96	0.91, 1.02	0.19	
Hispanic men* education, per year > 12	-0.47	-1.16, 0.22	0.18	0.99	0.91, 1.07	0.75	
Hispanic women* education, per year > 12	-0.76*	-1.37, -0.15	0.01	0.95	0.88, 1.02	0.16	
Observations (Number of id)	51,692 (24,526)			51,693 (24,526)			
F-test of joint significance for interactions	chi^2^ = 42.41 (*p* = 0.00)			chi^2^ = 53.31 (*p* = 0.00)			

This table shows coefficients from our interaction models on the race by gender subgroups. The main effects of the three components of the education spline (< 12 years, 12 years Y/N, > 12 years) represent associations for the reference group, White men, while the interaction terms show the differential associations for each of the race by gender subgroups. Each of the interaction terms represents the difference in the association between education and hypertension/blood pressure between the reference group (White men) and the other race*gender groups. For example, the interaction term White women*education, per year > 12 indicates that, compared to White men (for whom each year of education after 12 years is associated with 0.54 mmHg lower SBP), for White women, each additional year of schooling after 12 years is associated with an additional 0.33 mmHg lower SBP, for a total of 0.54 mmHg + 0.33 mmHg = 0.87 mmHg. Statistical significance is indicated as following: *** P-value < 0.001, ** P-value < 0.01, * P-value < 0.05. Regressions were adjusted for age, place of birth (foreign birth, southern US, non-southern US, or not specified), year of BP measurement (2006–2018), and mother’s and father’s education

Stratified results indicated that each year of education was associated with lower SBP and lower odds of hypertension among all the subgroups, but interaction analyses showed that there were some differences in the size of these associations. Compared to White men, for whom each additional year of education after 12 years was associated with a 0.54 mmHg lower SBP ( 95% CI=-0.74, -0.33) and 6% lower odds of having HTN (OR = 0.94, 95% CI = 0.92, 0.97), we found the following differences in cardiovascular risk: lower SBP for White women (interaction b=-0.33 mmHg, 95% CI=-0.61, -0.06), Black women (interaction b=-0.52 mmHg, 95% CI = 1.00, -0.04) and Hispanic women (interaction b=-0.76, CI=-1.37, -0.15), lower likelihood of HTN for White women (interaction OR = 0.96, 95% CI = 0.93, 1.00). Results from the F-test indicated that the interaction terms included in our model were jointly significant.

Results from stratified analyses (Figs. 2 and 3) showed that the association of 12 years of education with blood pressure went in the opposite direction for Black men (b = 1.20 mmHg, 95% CI=-1.86, 4.26) alone (e.g. White men, b=-2.00 mmHg, 95% CI=-4.19, 0.20). Stratified results also showed that each additional year of education after 12 years was associated with the smallest blood pressure reduction among Black men (b = 0.50 mmHg, 95% CI =-1.05, 0.03) and largest blood pressure reduction among Black women (b = 1.02 mmHg, 95% CI=-1.48, -0.57). A similar pattern was observed for hypertension.

### Robustness checks

Results from robustness checks including different operationalizations of the outcome (Tables S3 and S4, Figure S4), the exposure (Tables S5 and S6, Figure S5), using multiple imputations for missing data (Tables S7 and S8, Figure S6) and excluding non-US born participants (Table S9 and S10, Figure S7) were in the same direction as the main results. The main conclusions were unchanged, although statistical significance varied for some of the interaction terms.

## Discussion

Using a longitudinal dataset of US adults 50 + years, we found evidence of differential associations by race and gender between education and blood pressure. Overall, high school diploma and each additional year of schooling after 12 years were associated with lower systolic blood pressure and lower odds of hypertension. However, we found that a high school diploma was associated with poorer cardiovascular risk outcomes for Black men, a reversal of the most reported education-blood pressure relationship. The benefit from each additional year of schooling after 12 years was greater for White women, Black women, and Hispanic women compared to White men.

Our finding of worse blood pressure among Black men who finished high school is consistent with the Minorities’ Diminished Returns theory. This framework suggests that the protective effects of higher socioeconomic positions may be smaller – or reversed – for minoritized groups than White men, mainly due to racism and discrimination [29, 63]. Previous studies have found diminished health effects of education in Black men compared to White men, White women, and Black women [64]. A study using HRS data showed that school term length, a measure of educational quality, was associated with lower blood pressure for Black women, but not for Black men [27]. A longitudinal study among White and Black participants found higher depressive symptoms among Black men who had at least 12 years of education compared to those without a high school degree [63]. A possible explanation for our finding is that as higher educational attainment creates inroads for Black men to access more advantaged communities, they remain exposed to the reinvented, yet persistent, exclusionary mechanisms (e.g., structural, institutional, and interpersonal racial discrimination) operating within these spaces [63, 65, 66]. That is, once Black men achieve a higher socioeconomic position through education, they may be more exposed to unfair differences in occupational attainment and promotion, income acquisition and intentional or unintentional interpersonal racial discrimination, which may be associated with stress, high blood pressure and hypertension [67]. Discrimination may also happen at the healthcare system level, leading to sub-optimal hypertension prevention and treatment [68]. Prior research found that Black men are more likely to perceive poor treatment in the health care setting with subsequent reluctance to engage with the health care system resulting in suboptimal chronic disease screening, and delayed prescription filling [69–72].

Our findings that White women, Black women, and Hispanic women benefited more from each year of education after completing 12 years of schooling compared to White men is consistent with the Resource Substitution hypothesis [10, 73]. Women may have greater health benefits from education than men if they are otherwise excluded from other socioeconomic resources such as power, authority, and earnings due to sexism resulting in structural gender inequality.

Taken together, our results suggest that testing for effect modification at the intersection between gender and race/ethnicity is relevant to understanding the complexity of the relationship between education and blood pressure and its contribution to creating health inequities. This is in line with Intersectionality theory, which suggests that interlocking systems of oppression produce predictable patterns of population health inequity by race and gender [55]. The difference in the cardiovascular benefit of education among Black men relative to Black women particularly emphasizes this point: had we evaluated the association only by race, we may not have been able to detect a difference in the high school diploma effect.

Our study has several strengths. We used a large and racially diverse sample of older adults in the US. We evaluated two different predictors of cardiovascular risk (hypertension and systolic blood pressure), and repeated blood pressure measurements. Rather than simply controlling for race or gender in our statistical model, we evaluated whether the relationship between education and cardiovascular risk differs by race/ethnicity by gender considered jointly, which helps to document differential and unique patterns at the nexus of race and gender identities [74].

There were a few limitations to our study. Estimated associations may still reflect unmeasured or residual confounding factors, such as childhood characteristics. In addition, our analytical sample only includes older adults, and our findings may not be generalizable to a younger population. The generalized estimating equations model with unbalanced BP measurements across participants used in our analysis did not allow including sampling weights, therefore our results are not generalizable to the US population. We also acknowledge that years of schooling and antihypertensive medication use were obtained from participant self-report and may be subject to reporting bias [75]. Moreover, the HRS item on self-identified race and ethnicity was limited to White, Black, Hispanic or “other” in the public data due to confidentiality reasons. The lack of more granular racial and ethnic data may also result in potential biases due to collapsing groups subject to different experiences of structural racism [50]. Although we conducted multiple imputations for missing variables among eligible participants, we were not able to include participants who died or dropped out before 2006. If the rate of death or dropout differed according to the exposure or effect modifier, our study would be affected by selective survival, which could produce bias in the effect of education on blood pressure [76]. Finally, 12 years of education, which correspond to a high school diploma in the United States, may not be equivalent to the same diploma in other countries. Although the HRS includes birthplace, information on where education was obtained is not available. Prior evidence suggests that education achieved in other countries may be under-valued in the host society, resulting in an attenuated beneficial association between education and later-life health [77].

Overall, our results suggest that programs and policies to increase years of education attainment after 12 years may reduce systolic blood pressure and hypertension among all subgroups. In addition, our findings on race-by-gender differential associations between education and blood pressure suggest the existence of deep structural inequities by race and gender at several levels, which need to be addressed through an all-policies approach. Examples of such policies are affirmative action policies to expand access to higher education, policies that expand access to public health insurance, policies that protect the rights of workers (such as discrimination laws and increase of minimum wage), and criminal-legal policies that reduce the prosecution and incarceration of youth.

## Conclusion

This study builds upon previous work on cardiovascular health disparities by evaluating whether the relationship between educational attainment and blood pressure varies at the intersection of race and gender. We found a protective association between higher educational attainment and blood pressure overall, and that White women and Hispanic women benefited more from each year of education when compared with White men. However, completing high school was associated with higher blood pressure among Black men. These results may reflect greater exposure to racially hostile environments for Black men per additional year of education which may be detrimental to their cardiovascular health. Future research should investigate whether educational environments themselves, as well as their sequelae, may differentially shape the lived experiences of Black men to inform interventions. Potential mediators of differences in the education-hypertension relationship by race and gender, such as stress, workplace discrimination and inequities in anti-hypertensive treatment should be investigated in future work. The results of our study are an initial step toward understanding the complexities of cardiovascular health inequities and identifying the most vulnerable groups based on multiple social identities.

## Electronic supplementary material

Below is the link to the electronic supplementary material.

Supplementary Material 1

## Data Availability

All the data used in this study is publicly available through the Health and Retirement Study: https://hrs.isr.umich.edu/. The STATA code for the analysis is available from the authors upon request.

## Declarations

### Previous presentations

Society of Epidemiological Research (SER).

## Abbreviations

HTN: Hypertension
SBP: Systolic Blood Pressure
CVD: Cardiovascular Disease
SES: Socio-economic status
HRS: Health and Retirement Study
EFTF: Enhanced Face-to-Face
BP: Blood Pressure
QIC: Quasi-Likelihood Information Criterion
GEE: Generalized Estimating Equations
ACC: American College of Cardiology
AHA: American Heart Association
MICE: Multiple Imputations by Chained Equations
GED: General Educational Development
OR: Odds Ratio
CI: Confidence Interval
