# Supplementary material for: Differential associations between education and blood pressure by gender and race
Source: BMC Public Health. 2025 Jul 2;25:2278. doi: 10.1186/s12889-025-23409-5 (PMC12220582; doi:10.1186/s12889-025-23409-5)
Supplement: Supplementary file 1 — Supplementary Material 1 [file 12889_2025_23409_MOESM1_ESM.doc]

**Supplemental Material**

Table S1 – Coding for the education spline

| **School years** | **Education < 12 years** | **Education > 12 years** | **Education>=12 (yes/no)** | **% Observations** |
| --- | --- | --- | --- | --- |
|  |  |  |  |  |
| 5 | -7 | 0 | 0 | 5.12 |
| 6 | -6 | 0 | 0 | 2.37 |
| 7 | -5 | 0 | 0 | 1.79 |
| 8 | -4 | 0 | 0 | 5.29 |
| 9 | -3 | 0 | 0 | 3.63 |
|  |  |  |  |  |
| 10 | -2 | 0 | 0 | 5.22 |
| 11 | -1 | 0 | 0 | 5.03 |
| 12 | 0 | 0 | 1 | 31.2 |
| 13 | 0 | 1 | 1 | 7.06 |
| 14 | 0 | 2 | 1 | 9.96 |
|  |  |  |  |  |
| 15 | 0 | 3 | 1 | 3.68 |
| 16 | 0 | 4 | 1 | 10.09 |
| 17 | 0 | 5 | 1 | 9.24 |

This table shows the coding for the education spline, our exposure variable. This operationalization models education as two separate slopes (< 12 years of schooling and > 12 years of schooling) and an indicator variable capturing a potential discontinuity at 12 years of schooling (which corresponds to achieving a high school diploma).

**Appendix 1. Supplemental Methods, Choice of functional form for education**

The functional form for education, our exposure, was chosen based on the following steps:

1. We looked at the distribution of systolic blood pressure (SBP) and hypertension (HTN) prevalence across years of schooling, and observed potential discontinuity at 11, 12 or 13 years of education (Figures A1 and A2).
2. We ran our base model on the association between education and SBP/HTN for linear years of schooling the three potential discontinuity years (11, 12 and 13) and terminal degree (less than high school/GED, high school or college) and we compared the quasi-likelihood information criterion (QIC) to select the best model fit (Table A1).

In addition to best fitting model, our choice was also motivated by interpretation, since 12 years corresponds to the typical high school degree year.

**Figure S1 – SBP and HTN prevalence across years of schooling**


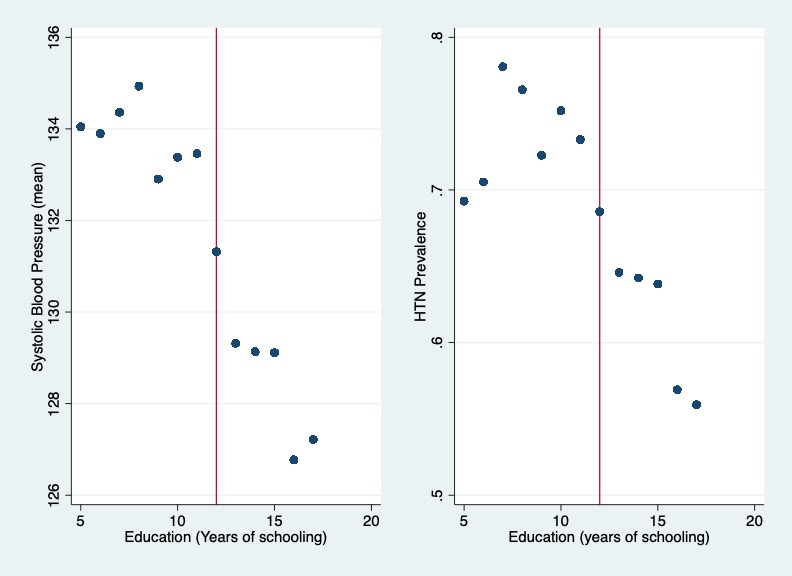


Education was operationalized as self-reported years of schooling (0-17). Due to data sparseness, respondents with fewer than 5 years of schooling were recoded to 5 years. Hypertension (HTN) was defined as either having blood pressure >= 140/90 mmHg, or taking hypertensive medications. Both mean SBP and hypertension are obtained from repeated observations from 2006-2018.

**Table S2 – QIC criterion to identify the functional form for education**

|  | **SBP** | **HTN** |
| --- | --- | --- |
| School Years (Linear) | 19667508 | 99699 |
| Spine with knot at 10 years | 19656032 | 62520 |
| Spine with knot at 11 years | 19655607 | 62520 |
| Spine with knot at 12 years | 19655095 | 62521 |
| Degree | 19762863 | 62815 |
| Note: Quasi-information criterion (QIC) was obtained from generalized estimating equations estimating the association between each functional form for education and SBP/HTN. In each model, we adjusted for age, birth place, mother’s and father’s education, missing indicators for mother’s and father’s education, birthplace and year of outcome measurement. Lower QIC indicates better model fit. | | |
|  |  |  |

**Figure S2 – Systolic Blood Pressure, key interaction terms**

**
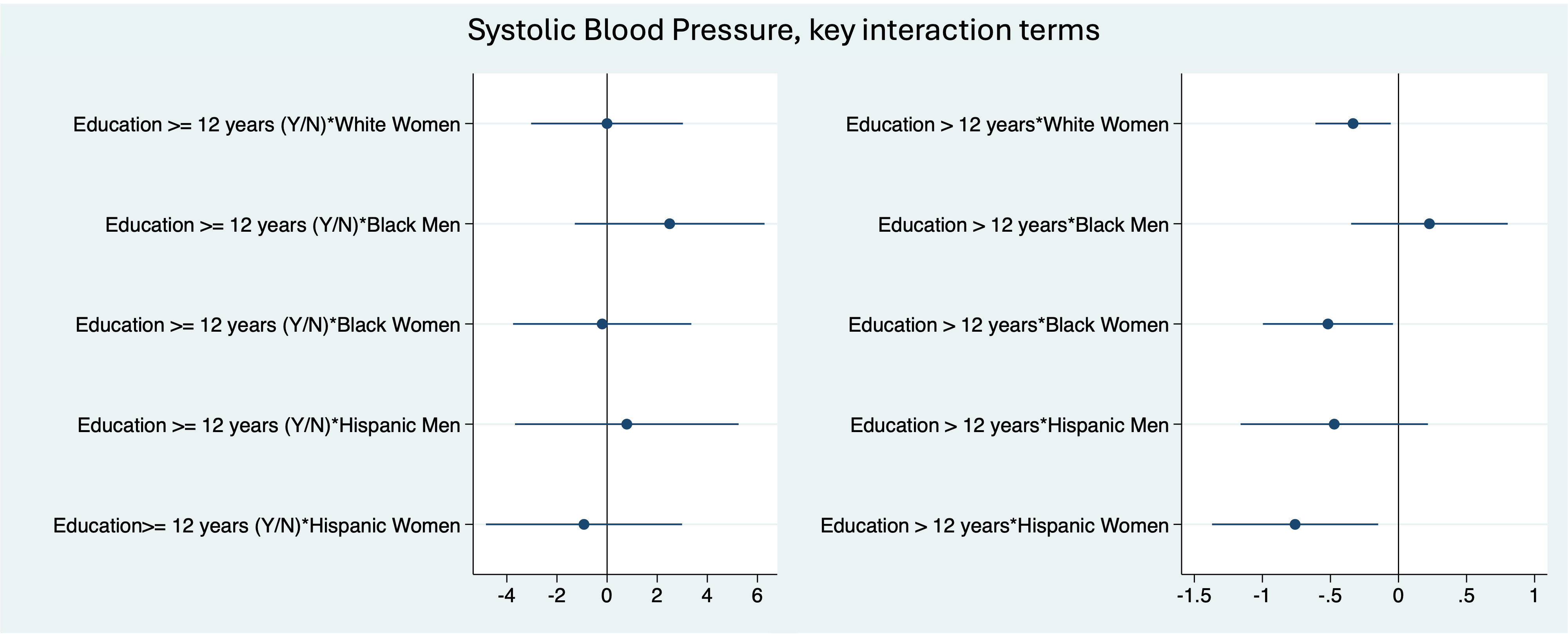
**

This figure shows beta coefficients on the interaction terms of interest in the GEE model estimating the relationship between education and systolic blood pressure (SBP). The left panel shows interactions between education >= 12 years (yes/no), race and gender, with White men as a reference group, which indicate the differences in the associations between 12 years of education and SBP between race-by-gender groups. The right panel shows interactions between education > 12 years), race and gender, with White men as a reference group, which indicate the differences in the associations between each additional year of education after 12 years and SBP.

**Figure S3 – Hypertension, key interaction terms**

**
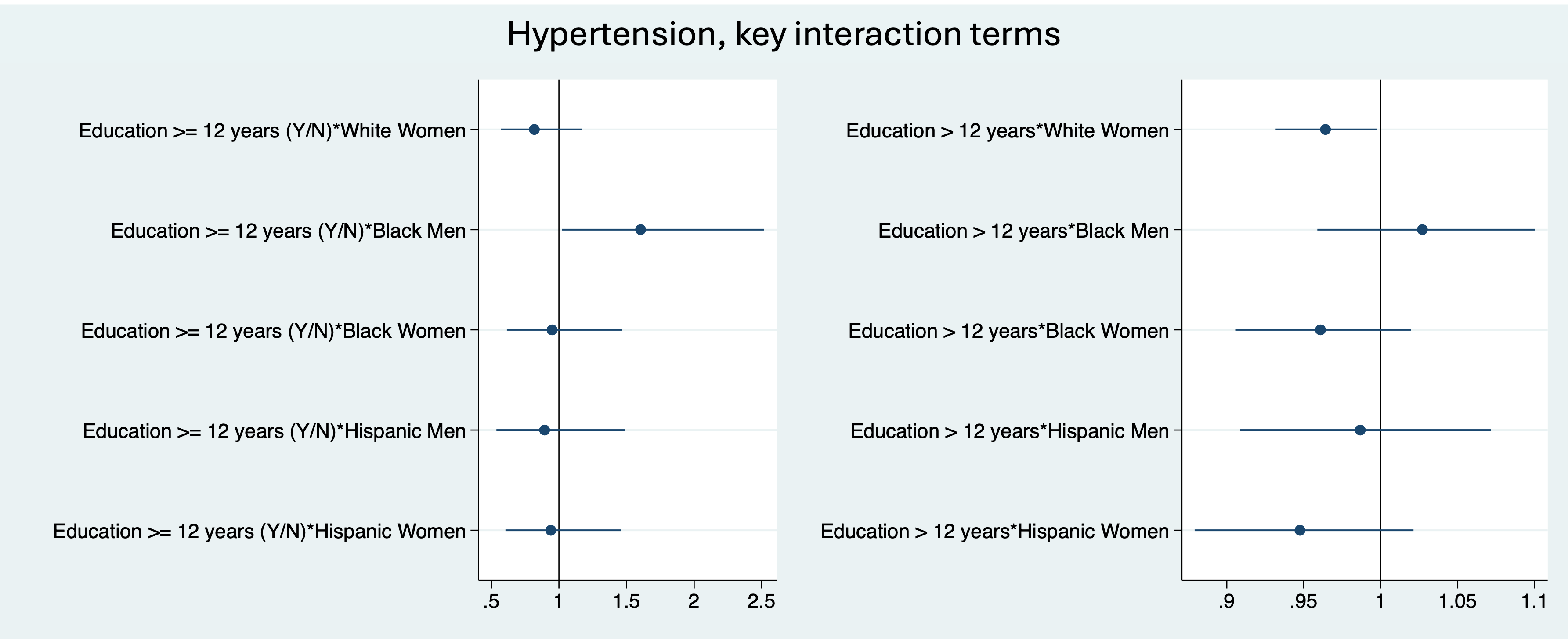
**

This figure shows odds ratios on the interaction terms of interest in the GEE model estimating the relationship between education and hypertension (HTN). The left panel shows interactions between education >= 12 years (yes/no), race and gender, with White men as a reference group, which indicate the differences in the associations between 12 years of education and HTN risk between race-by-gender groups. The right panel shows interactions between education > 12 years, race and gender, with White men as a reference group, which indicate the differences in the associations between each additional year of education after 12 years and HTN risk.

**Table S3 – GEE, base model with diastolic BP and hypertension based on 2017 guidelines as outcomes**

|  | **Diastolic Blood Pressure** | | | **Hypertension** | | | |  |
| --- | --- | --- | --- | --- | --- | --- | --- | --- |
| VARIABLES | β | 95% CI | P-value | Odds ratio | | 95% CI | P-value |  |
| Education, per year < 12 | 0.12 | -0.02, 0.26 | 0.09 | 1.01 | | 0.98, 1.05 | 0.38 |  |
| Education >=12 years (Y/N) | -0.66* | -1.25, -0.08 | 0.03 | 0.86* | | 0.75, 0.98 | 0.03 |  |
| Education, per year > 12 | -0.28*** | -0.35, -0.20 | 0.00 | 0.92*** | | 0.91, 0.94 | 0.00 |  |
| Race and Ethnicity (Ref=White) |  |  |  |  | |  |  |  |
| Black | 2.75*** | 2.37, 3.12 | 0.00 | 2.26*** | | 2.07, 2.47 | 0.00 |  |
| Hispanic | 0.20 | -0.30, 0.70 | 0.43 | 1.20*** | | 1.08, 1.34 | 0.00 |  |
| Gender (Ref=Male) |  |  |  |  | |  |  |  |
| Female | -0.69*** | -0.94, -0.44 | 0.00 | 0.74*** | | 0.70, 0.79 | 0.00 |  |
| Father's Education | 0.01 | -0.04, 0.05 | 0.81 | 0.99* | | 0.98, 1.00 | 0.01 |  |
| Mother's Education | 0.07** | 0.02, 0.12 | 0.00 | 1.00 | | 0.99, 1.01 | 0.45 |  |
| Missing Mother's Education | 0.05 | -0.46, 0.56 | 0.84 | 0.97 | | 0.87, 1.09 | 0.66 |  |
| Missing Father's Education | 0.24 | -0.17, 0.64 | 0.25 | 1.12* | | 1.02, 1.22 | 0.02 |  |
| Birthplace (Ref. = Non-Southern US) |  |  |  |  | |  |  |  |
| Southern birth | 0.04 | -0.25, 0.34 | 0.77 | 1.20*** | | 1.12, 1.28 | 0.00 |  |
| Immigrant | -0.63* | -1.11, -0.14 | 0.01 | 0.83*** | | 0.75, 0.91 | 0.00 |  |
| US not specified | 0.95* | 0.06, 1.84 | 0.04 | 1.17 | | 0.96, 1.44 | 0.12 |  |
| Age | -0.22*** | -0.23, -0.21 | 0.00 | 1.03*** | | 1.03, 1.04 | 0.00 |  |
| Year (Ref=2006) |  |  |  |  | |  |  |  |
| 2008 | 0.16 | -0.22, 0.53 | 0.41 | 1.08 | | 1.00, 1.17 | 0.06 |  |
| 2010 | 0.35* | 0.05, 0.65 | 0.02 | 1.15*** | | 1.09, 1.22 | 0.00 |  |
| 2012 | -1.00*** | -1.36, -0.64 | 0.00 | 1.00 | | 0.93, 1.08 | 0.91 |  |
| 2014 | -1.25*** | -1.57, -0.93 | 0.00 | 0.97 | | 0.91, 1.04 | 0.40 |  |
| 2016 | -1.93*** | -2.29, -1.57 | 0.00 | 0.92* | | 0.86, 0.99 | 0.03 |  |
| 2018 | -2.29*** | -2.63, -1.95 | 0.00 | 0.93* | | 0.87, 1.00 | 0.04 |  |
| Constant | 94.82*** | 93.64, 96.01 | 0.00 | 0.56*** | | 0.43, 0.73 | 0.00 |  |
|  |  |  |  |  | |  |  |  |
| Observations | 51,692 (24,526) | | | | 51,693 (24,526) | | | |

Education is operationalized as a spline using self-reported years of schooling (5-17). Mother’s and father’s education are parents’ years of schooling, and missing mother’s and father’s education are indicator variables. Statistical significance is indicated as following: *** P-value <0.001, ** P-value <0.01, * P-value <0.05.

**Table S4 – GEE, interaction model with diastolic BP and hypertension based on 2017 guidelines as outcomes**

|  | | **Diastolic Blood Pressure** | | | | | | | | | | | **Hypertension** | | | | | | | | | | |  |
| --- | --- | --- | --- | --- | --- | --- | --- | --- | --- | --- | --- | --- | --- | --- | --- | --- | --- | --- | --- | --- | --- | --- | --- | --- |
| VARIABLES | β | | | | | 95% CI | | | P-value | | | | | Odds Ratio | | | 95% CI | | | P-value | | | | |
| Education, per year < 12 | | | 0.06 | | -0.29, 0.41 | | | 0.74 | | | 1.00 | | | | | 0.92, 1.09 | | | 0.97 | | |  | | |
| White women | | -0.55 | | -2.20, 1.09 | | | 0.51 | | | 0.94 | | | | | 0.64, 1.39 | | | 0.77 | | |  | | | |
| Black men | | 2.39* | | 0.30, 4.49 | | | 0.03 | | | 1.50 | | | | | 0.91, 2.47 | | | 0.11 | | |  | | | |
| Black women | | 3.29*** | | 1.40, 5.18 | | | 0.00 | | | 2.50*** | | | | | 1.56, 4.01 | | | 0.00 | | |  | | | |
| Hispanic men | | 1.33 | | -0.97, 3.63 | | | 0.26 | | | 1.57 | | | | | 0.91, 2.70 | | | 0.11 | | |  | | | |
| Hispanic women | | -0.59 | | -2.63, 1.44 | | | 0.57 | | | 0.96 | | | | | 0.61, 1.51 | | | 0.85 | | |  | | | |
| White women*education, per year < 12 | | -0.11 | | -0.62, 0.40 | | | 0.68 | | | 1.02 | | | | | 0.90, 1.15 | | | 0.81 | | |  | | | |
| Black men*education, per year < 12 | | -0.07 | | -0.66, 0.52 | | | 0.81 | | | 0.97 | | | | | 0.85, 1.12 | | | 0.71 | | |  | | | |
| Black women*education, per year < 12 | | 0.25 | | -0.30, 0.80 | | | 0.37 | | | 1.06 | | | | | 0.92, 1.22 | | | 0.43 | | |  | | | |
| Hispanic men*education, per year < 12 | | 0.15 | | -0.36, 0.66 | | | 0.57 | | | 1.06 | | | | | 0.94, 1.19 | | | 0.32 | | |  | | | |
| Hispanic women*education, per year < 12 | | 0.04 | | -0.42, 0.50 | | | 0.86 | | | 0.99 | | | | | 0.89, 1.10 | | | 0.83 | | |  | | | |
|  | |  | |  | | |  | | |  | | | | |  | | |  | | |  | | | |
| Knot (12 years) | | -0.75 | | -2.01, 0.51 | | | 0.24 | | | 0.96 | | | | | 0.71, 1.30 | | | 0.80 | | |  | | | |
| White women*education >=12 years (Y/N) | | 0.48 | | -1.24, 2.19 | | | 0.58 | | | 0.79 | | | | | 0.53, 1.19 | | | 0.26 | | |  | | | |
| Black men*education >=12 years (Y/N) | | 1.25 | | -1.01, 3.51 | | | 0.28 | | | 1.28 | | | | | 0.75, 2.20 | | | 0.37 | | |  | | | |
| Black women*education >=12 years (Y/N) | | -0.51 | | -2.52, 1.50 | | | 0.62 | | | 0.85 | | | | | 0.51, 1.41 | | | 0.53 | | |  | | | |
| Hispanic men*education >=12 years (Y/N) | | 0.51 | | -2.02, 3.04 | | | 0.69 | | | 0.84 | | | | | 0.47, 1.52 | | | 0.57 | | |  | | | |
| Hispanic women*education >=12 years (Y/N) | | 0.24 | | -1.97, 2.45 | | | 0.83 | | | 0.88 | | | | | 0.54, 1.44 | | | 0.62 | | |  | | | |
| Education, per year > 12 | | -0.15* | | -0.27, -0.02 | | | 0.02 | | | 0.94*** | | | | | 0.92, 0.97 | | | 0.00 | | |  | | | |
| White women*education, per year > 12 | | -0.11 | | -0.27, 0.06 | | | 0.19 | | | 0.97 | | | | | 0.93, 1.00 | | | 0.07 | | |  | | | |
| Black men*education, per year > 12 | | -0.18 | | -0.53, 0.16 | | | 0.29 | | | 1.01 | | | | | 0.94, 1.10 | | | 0.72 | | |  | | | |
| Black women*education, per year > 12 | | -0.39** | | -0.66, -0.11 | | | 0.01 | | | 0.92* | | | | | 0.86, 0.98 | | | 0.01 | | |  | | | |
| Hispanic men*education, per year > 12 | | -0.15 | | -0.59, 0.29 | | | 0.51 | | | 1.01 | | | | | 0.92, 1.11 | | | 0.80 | | |  | | | |
| Hispanic women*education, per year > 12 | | -0.36 | | -0.73, 0.01 | | | 0.05 | | | 0.97 | | | | | 0.90, 1.05 | | | 0.45 | | |  | | | |
| Constant | | 95.41*** | | 93.64, 97.17 | | | 0.00 | | | 0.58** | | | | | 0.39, 0.87 | | | 0.01 | | |  | | | |
| Observations | | 51,692 (24,526) | | | | | | | | | | 51,693 (24,526) | | | | | | | | | | |  | |

Regressions are adjusted for age, birthplace, mother’s and father’s education, missing indicators for mother’s and father’s education, birthplace and year of outcome measurement. Education is operationalized as a spline using self-reported years of schooling (5-17). White men is the reference group for race and ethnicity*gender categories. Statistical significance is indicated as following: *** P-value <0.001, ** P-value <0.01, * P-value <0.05.

**Figure S4 – Stratified results with diastolic BP and hypertension based on 2017 guidelines as outcomes**


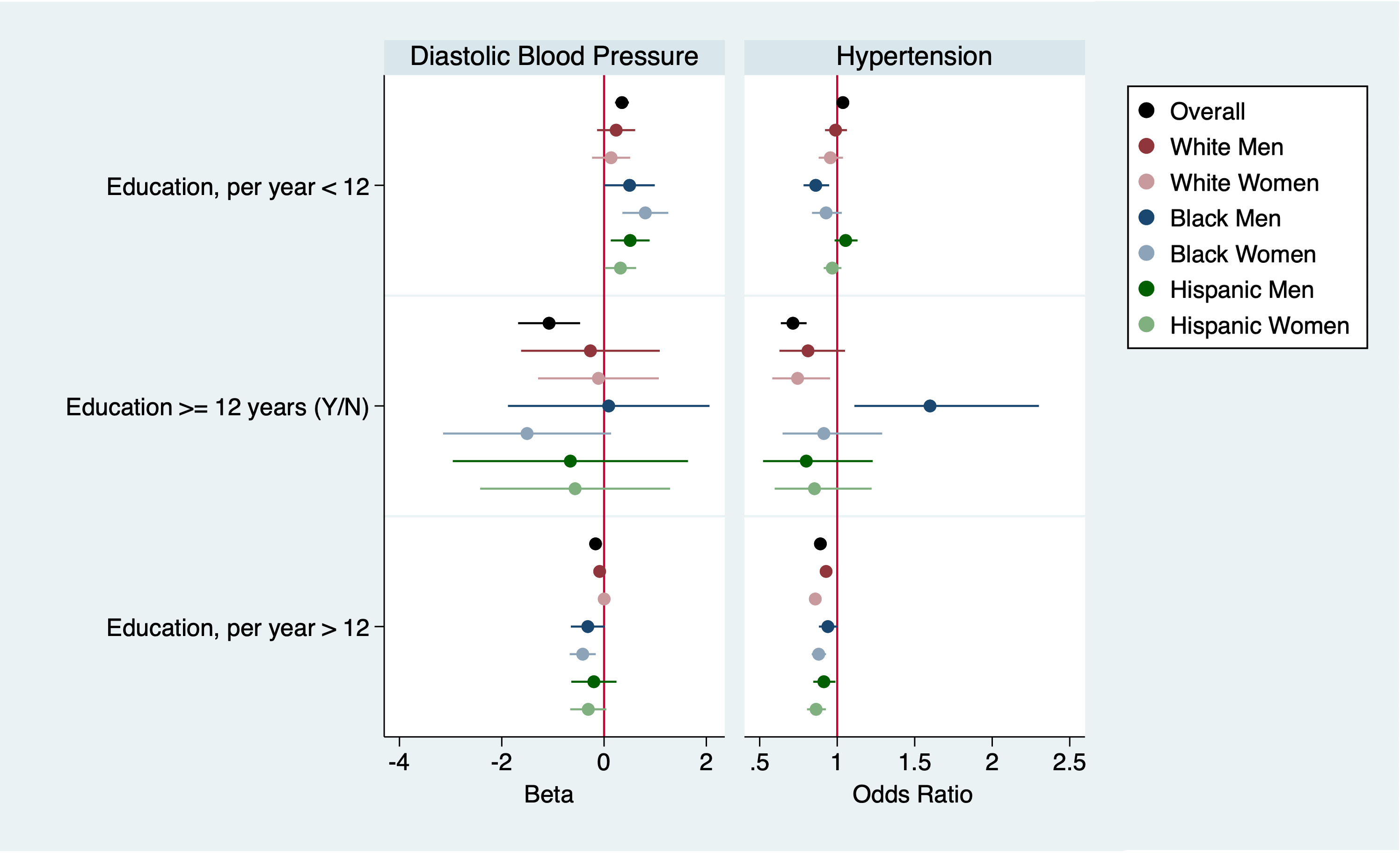


Regressions are adjusted for age, birthplace, mother’s and father’s education, missing indicators for mother’s and father’s education, birthplace and year of outcome measurement. Education is operationalized as a spline using self-reported years of schooling (5-17). Coefficient plots indicate beta coefficients for diastolic blood pressure and odds ratios for hypertension.

**Table S5 – Generalized estimating equations**, base model with terminal degree es exposure

|  | **Systolic Blood Pressure** | | | | | **Hypertension** | | | |
| --- | --- | --- | --- | --- | --- | --- | --- | --- | --- |
| VARIABLES | β | | 95% CI | P-value | | Odds ratio | 95% CI | P-value | |
| Degree (Ref=High School) |  | |  |  | |  |  |  | |
| < High School | 1.54*** | | 0.94, 2.14 | 0.00 | | 1.09* | 1.02, 1.17 | 0.02 | |
| College | -2.24*** | | -2.74, -1.74 | 0.00 | | 0.77*** | 0.73, 0.82 | 0.00 | |
| Race and Ethnicity (Ref=White) |  | |  |  | |  |  |  | |
| Black | 5.52*** | | 4.87, 6.17 | 0.00 | | 2.36*** | 2.18, 2.55 | 0.00 | |
| Hispanic | 1.68*** | | 0.83, 2.53 | 0.00 | | 1.20*** | 1.09, 1.32 | 0.00 | |
| Gender (Ref=Male) |  | |  |  | |  |  |  | |
| Female | -4.78*** | | -5.21, -4.36 | 0.00 | | 0.85*** | 0.81, 0.89 | 0.00 | |
| Father's Education | -0.10** | | -0.18, -0.03 | 0.01 | | 0.98*** | 0.97, 0.99 | 0.00 | |
| Mother's Education | 0.01 | | -0.07, 0.09 | 0.84 | | 1.00 | 0.99, 1.01 | 0.75 | |
| Missing Mother's Education | 0.38 | | -0.52, 1.29 | 0.41 | | 0.96 | 0.87, 1.07 | 0.46 | |
| Missing Father's Education | 0.19 | | -0.50, 0.89 | 0.59 | | 1.14** | 1.05, 1.23 | 0.00 | |
| Birthplace |  | |  |  | |  |  |  | |
| Southern birth | 0.95*** | | 0.45, 1.46 | 0.00 | | 1.18*** | 1.11, 1.26 | 0.00 | |
| Immigrant | -0.20 | | -1.01, 0.62 | 0.64 | | 0.81*** | 0.73, 0.88 | 0.00 | |
| US not specified | 0.72 | | -0.77, 2.20 | 0.34 | | 1.16 | 0.97, 1.39 | 0.10 | |
| Age | 0.31*** | | 0.29, 0.33 | 0.00 | | 1.05*** | 1.04, 1.05 | 0.00 | |
| Year (Ref=2006) |  | |  |  | |  |  |  | |
| 2008 | 0.02 | | -0.63, 0.66 | 0.96 | | 1.02 | 0.95, 1.09 | 0.65 | |
| 2010 | 0.49 | | -0.02, 1.00 | 0.06 | | 1.12*** | 1.06, 1.17 | 0.00 | |
| 2012 | -1.87*** | | -2.48, -1.25 | 0.00 | | 1.04 | 0.97, 1.11 | 0.27 | |
| 2014 | -2.34*** | | -2.89, -1.79 | 0.00 | | 1.03 | 0.97, 1.08 | 0.32 | |
| 2016 | -2.90*** | | -3.51, -2.28 | 0.00 | | 0.95 | 0.89, 1.02 | 0.15 | |
| 2018 | -3.70*** | | -4.29, -3.11 | 0.00 | | 0.97 | 0.91, 1.03 | 0.31 | |
| Constant | 112.64*** | | 110.80, 114.48 | 0.00 | | 0.11*** | 0.09, 0.14 | 0.00 | |
|  |  | |  |  | |  |  |  | |
| Observations (Number of id) | | 51,876 (24,619) | | | 51,876 (24,619) | | | |  |

Mother’s and father’s education are parents’ years of schooling, and missing mother’s and father’s education are indicator variables. Statistical significance is indicated as following: *** P-value <0.001, ** P-value <0.01, * P-value <0.05.

**Table S6 – Generalized estimating equations**, interaction model with terminal degree es exposure

|  | **Systolic Blood Pressure** | | | **Hypertension** | | | |  |
| --- | --- | --- | --- | --- | --- | --- | --- | --- |
| VARIABLES | β | 95% CI | P-value | Odds Ratio | | 95% CI | P-value |  |
| Degree (Ref=High School) |  |  |  |  | |  |  |  |
| < High School/GED | 1.60** | 0.45, 2.75 | 0.01 | 1.11 | | 0.96, 1.27 | 0.15 |  |
| College | -1.28** | -2.11, -0.46 | 0.00 | 0.82*** | | 0.74, 0.91 | 0.00 |  |
| Race and Ethnicity*Gender (Ref=White men) |  |  |  |  | |  |  |  |
| White women | -4.03*** | -4.78, -3.28 | 0.00 | 0.82*** | | 0.75, 0.90 | 0.00 |  |
| Black men | 5.64*** | 4.24, 7.04 | 0.00 | 2.11*** | | 1.78, 2.51 | 0.00 |  |
| Black women | 1.63* | 0.39, 2.86 | 0.01 | 2.20*** | | 1.89, 2.55 | 0.00 |  |
| Hispanic men | 4.05*** | 2.35, 5.75 | 0.00 | 1.44*** | | 1.18, 1.77 | 0.00 |  |
| Hispanic women | -4.08*** | -5.58, -2.57 | 0.00 | 1.01 | | 0.85, 1.21 | 0.87 |  |
| < High School/GED*White women | 0.17 | -1.39, 1.72 | 0.83 | 1.12 | | 0.93, 1.34 | 0.24 |  |
| < High School/GED*Black men | -1.61 | -3.83, 0.60 | 0.15 | 0.69** | | 0.53, 0.91 | 0.01 |  |
| < High School/GED *Black women | 0.07 | -1.98, 2.11 | 0.95 | 1.03 | | 0.80, 1.32 | 0.83 |  |
| < High School/GED *Hispanic men | -1.54 | -3.85, 0.77 | 0.19 | 0.72* | | 0.55, 0.94 | 0.02 |  |
| < High School/GED*Hispanic women | 1.94 | -0.13, 4.00 | 0.07 | 1.06 | | 0.83, 1.34 | 0.65 |  |
|  |  |  |  |  | |  |  |  |
| College*White women | -1.68** | -2.79, -0.57 | 0.00 | 0.88 | | 0.77, 1.01 | 0.08 |  |
| College*Black men | 0.70 | -1.49, 2.88 | 0.53 | 1.08 | | 0.83, 1.40 | 0.58 |  |
| College*Black women | -1.53 | -3.36, 0.29 | 0.10 | 0.95 | | 0.76, 1.18 | 0.63 |  |
| College*Hispanic men | -1.87 | -4.38, 0.64 | 0.15 | 0.81 | | 0.59, 1.10 | 0.18 |  |
| College*Hispanic women | -1.88 | -4.18, 0.43 | 0.11 | 0.79 | | 0.59, 1.04 | 0.10 |  |
| Constant | 112.08*** | 110.19, 113.96 | 0.00 | 0.11*** | | 0.09, 0.14 | 0.00 |  |
|  |  |  |  |  | |  |  |  |
| Participants (Observations) | 24,619 (51,876) | | | | 24,619 (51,876) | | | |

Regressions are adjusted for age, birthplace, mother’s and father’s education, missing indicators for mother’s and father’s education, birthplace and year of outcome measurement. Statistical significance is indicated as following: *** P-value <0.001, ** P-value <0.01, * P-value <0.05.

**Figure S5 – Stratified results with terminal degree es exposure**


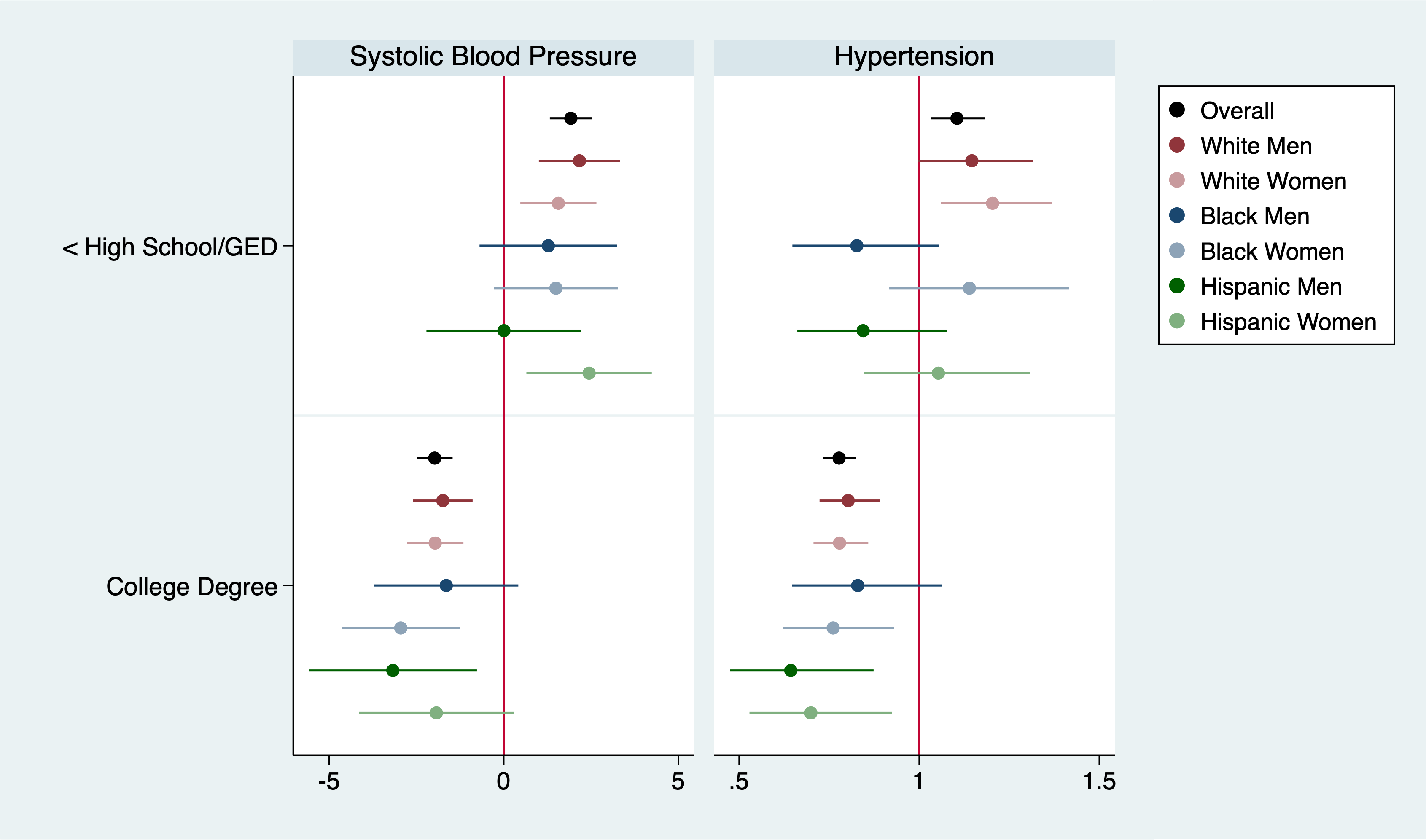


Terminal degree is operationalized as a categorical variable: <high school/GED, high school or college degree. High school degree is set as reference group. Regressions are adjusted for age, birthplace, mother’s and father’s education, missing indicators for mother’s and father’s education, birthplace and year of outcome measurement. Coefficient plots indicate beta coefficients for systolic blood pressure and odds ratios for hypertension.

**Table S7 – Generalized Estimating Equation, base models using imputed datasets for outcomes**

|  | **Systolic Blood Pressure** | | | | | | | | | | **Hypertension** | | | | | | | |
| --- | --- | --- | --- | --- | --- | --- | --- | --- | --- | --- | --- | --- | --- | --- | --- | --- | --- | --- |
| VARIABLES | β | | | 95% CI | | | P-value | | | | Odds ratio | | 95% CI | | | P-value | | |
| Education < 12 years | -0.15 | | | -0.40, 0.09 | | | 0.22 | | | | 1.00 | | 0.97, 1.02 | | | 0.73 | | |
| Education >=12 years (Y/N) | -0.85 | | | -1.83, 0.13 | | | 0.09 | | | | 0.91 | | 0.81, 1.02 | | | 0.09 | | |
| Education > 12 years | -0.72*** | | | -0.85, -0.59 | | | 0.00 | | | | 0.93*** | | 0.92, 0.94 | | | 0.00 | | |
| Race and Ethnicity (Ref=White) |  | | |  | | |  | | | |  | |  | | |  | | |
| Black | | | 4.73*** | | | 4.10, 5.36 | | | 0.00 | | | 2.25*** | | | 2.10, 2.42 | | | 0.00 |
| Hispanic | | | 1.82*** | | | 0.97, 2.66 | | | 0.00 | | | 1.20*** | | | 1.10, 1.32 | | | 0.00 |
| Gender (Ref=Male) |  | | |  | | |  | | | |  | |  | | |  | | |
| Female | -4.80*** | | | -5.26, -4.35 | | | 0.00 | | | | 0.84*** | | 0.80, 0.89 | | | 0.00 | | |
| Father’s education | -0.09* | | | -0.16, 0.01 | | | 0.03 | | | | 0.99*** | | 0.98, 1.00 | | | 0.00 | | |
| Mother’s education | 0.03 | | | -0.05, 0.11 | | | 0.40 | | | | 1.00 | | 0.99, 1.01 | | | 0.75 | | |
| Missing mother’s education | 0.18 | | | -0.71, 1.08 | | | 0.69 | | | | 0.95 | | 0.87, 1.05 | | | 0.33 | | |
| Missing father’s education | 0.21 | | | -0.49, 0.92 | | | 0.55 | | | | 1.10* | | 1.02, 1.19 | | | 0.01 | | |
| Birthplace (Ref=Non-Southern US) |  | | |  | | |  | | | |  | |  | | |  | | |
| Southern birth | 1.14*** | | | 0.63, 1.64 | | | 0.00 | | | | 1.21*** | | 1.15, 1.29 | | | 0.00 | | |
| Immigrant | -0.45 | | | -1.27, 0.37 | | | 0.28 | | | | 0.81*** | | 0.74, 0.88 | | | 0.00 | | |
| US not specified | 0.91 | | | -0.62, 2.44 | | | 0.25 | | | | 1.18 | | 1.00, 1.39 | | | 0.05 | | |
| Age  Wave (Ref=2006/2008) | -0.31*** | | | 0.29, 0.33 | | | 0.00 | | | | 1.05*** | | 1.04, 1.05 | | | 0.00 | | |
| 2010/2012 | -0.57*** | | | -0.95, -0.18 | | | 0.00 | | | | 1.06*** | | 1.03, 1.10 | | | 0.00 | | |
| 2014/2016 | | -2.57*** | | | -3.01, -2.12 | | | 0.00 | | 0.96 | | | | 0.93, 1.00 | | | 0.05 | |
| 2018 | | -4.16*** | | | -4.71, -3.61 | | | 0.00 | | 0.85*** | | | | 0.81, 0.90 | | | 0.00 | |
|  |  | | |  | | |  | | | |  | |  | | |  | | |
| Constant | 114.00*** | | | 111.93, 116.07 | | | 0.00 | | | | 0.13*** | | 0.10, 0.16 | | | 0.00 | | |
|  |  | | |  | | |  | | | |  | |  | | |  | | |
| Participants (Observations) | 28,227 (66,091) | | | | | | | | | | 28,227 (66,091) | | | | | | | |

Missing outcomes, exposure, and covariates (except for mother’s and father’s education) were imputed using multiple imputations by chained equations with 30 iterations. Education is operationalized as a spline using self-reported years of schooling (5-17). Mother’s and father’s education are parents’ years of schooling, and missing mother’s and father’s education are indicator variables. Statistical significance is indicated as following: *** P-value <0.001, ** P-value <0.01, * P-value <0.05.

**Table S8 – Generalized Estimating Equation, interaction model using imputed datasets for outcomes**

|  | | **Systolic Blood Pressure** | | | | | | **Hypertension** | | | | | | |  | |
| --- | --- | --- | --- | --- | --- | --- | --- | --- | --- | --- | --- | --- | --- | --- | --- | --- |
| VARIABLES | β | | | | 95% CI | P-value | | | | Odds Ratio | 95% CI | | P-value | | | |
| Education, per year < 12 | | | 0.08 | -0.51, 0.67 | | | 0.79 | | 1.01 | | | 0.94, 1.09 | | 0.74 | |  |
| White women | | | -4.66*** | -7.49, -1.83 | | | 0.00 | | 0.91 | | | 0.65, 1.27 | | 0.58 | |  |
| Black men | | | 2.28 | -1.07, 5.63 | | | 0.18 | | 1.17 | | | 0.78, 1.75 | | 0.44 | |  |
| Black women | | | 0.83 | -2.42, 4.08 | | | 0.62 | | 2.30*** | | | 1.54, 3.42 | | 0.00 | |  |
| Hispanic men | | | 2.90 | -1.10, 6.91 | | | 0.16 | | 1.30 | | | 0.83, 2.05 | | 0.25 | |  |
| Hispanic women | | | -2.91 | -6.41, 0.58 | | | 0.10 | | 0.94 | | | 0.64, 1.39 | | 0.77 | |  |
|  | | |  |  | | |  | |  | | |  | |  | |  |
| White women*education, per year < 12 | | | -0.32 | -1.18, 0.55 | | | 0.47 | | 0.96 | | | 0.86, 1.06 | | 0.41 | |  |
| Black men*education, per year < 12 | | | -0.36 | -1.29, 0.56 | | | 0.44 | | 0.91 | | | 0.81, 1.02 | | 0.11 | |  |
| Black women*education, per year < 12 | | | -0.01 | -0.97, 0.95 | | | 0.99 | | 1.02 | | | 0.90, 1.15 | | 0.76 | |  |
| Hispanic men*education, per year < 12 | | | -0.05 | -0.89, 0.78 | | | 0.90 | | 1.03 | | | 0.93, 1.14 | | 0.56 | |  |
| Hispanic women*education, per year < 12 | | | -0.28 | -1.07, 0.50 | | | 0.48 | | 0.95 | | | 0.87, 1.04 | | 0.27 | |  |
| Knot (12 years) | | | -1.39 | -3.58, 0.79 | | | 0.21 | | 0.91 | | | 0.70, 1.19 | | 0.49 | |  |
| White women*knot (12 years) | | | 0.34 | -2.62, 3.31 | | | 0.82 | | 0.90 | | | 0.63, 1.27 | | 0.55 | |  |
| Black men*knot (12 years) | | | 2.40 | -1.18, 5.99 | | | 0.19 | | 1.68* | | | 1.09, 2.60 | | 0.02 | |  |
| Black women*knot (12 years) | | | 0.11 | -3.34, 3.57 | | | 0.95 | | 0.93 | | | 0.61, 1.42 | | 0.75 | |  |
| Hispanic men*knot (12 years) | | | 0.67 | -3.65, 4.99 | | | 0.76 | | 0.99 | | | 0.60, 1.63 | | 0.97 | |  |
| Hispanic women*knot (12 years) | | | -0.43 | -4.24, 3.37 | | | 0.82 | | 1.05 | | | 0.68, 1.61 | | 0.83 | |  |
|  | | |  |  | | |  | |  | | |  | |  | |  |
| Education, per year > 12 | | | -0.54*** | -0.75, -0.33 | | | 0.00 | | 0.95*** | | | 0.92, 0.97 | | 0.00 | |  |
| White women*education, per year > 12 | | | -0.27 | -0.55, 0.01 | | | 0.06 | | 0.97* | | | 0.93, 1.00 | | 0.04 | |  |
| Black men*education, per year > 12 | | | 0.14 | -0.42, 0.71 | | | 0.62 | | 1.01 | | | 0.94, 1.08 | | 0.86 | |  |
| Black women*education, per year > 12 | | | -0.43 | -0.89, 0.02 | | | 0.06 | | 0.97 | | | 0.92, 1.02 | | 0.26 | |  |
| Hispanic men*education, per year > 12 | | | -0.38 | -1.07, 0.31 | | | 0.28 | | 1.00 | | | 0.92, 1.08 | | 0.99 | |  |
| Hispanic women*education, per year > 12 | | | -0.57 | -1.21, 0.07 | | | 0.08 | | 0.95 | | | 0.89, 1.02 | | 0.17 | |  |
|  | | |  |  | | |  | |  | | |  | |  | |  |
| Constant | | | 114.08*** | 111.28, 116.88 | | | 0.00 | | 0.13*** | | | 0.09, 0.18 | | 0.00 | |  |
| Participants (Observations) | | 28,227 (66,091) | | | | | | 28,227 (66,091) | | | | | | |  | |

Missing outcomes, exposure, and covariates (except for mother’s and father’s education) were imputed using multiple imputations by chained equations with 30 iterations. Regressions are adjusted for birth year, birthplace, mother’s and father’s education, missing indicators for mother’s and father’s education, birthplace and wave of outcome measurement. Education is operationalized as a spline using self-reported years of schooling (5-17). Statistical significance is indicated as following: *** P-value <0.001, ** P-value <0.01, * P-value <0.05.

**Figure S6 – Stratified results using imputed datasets for outcomes**


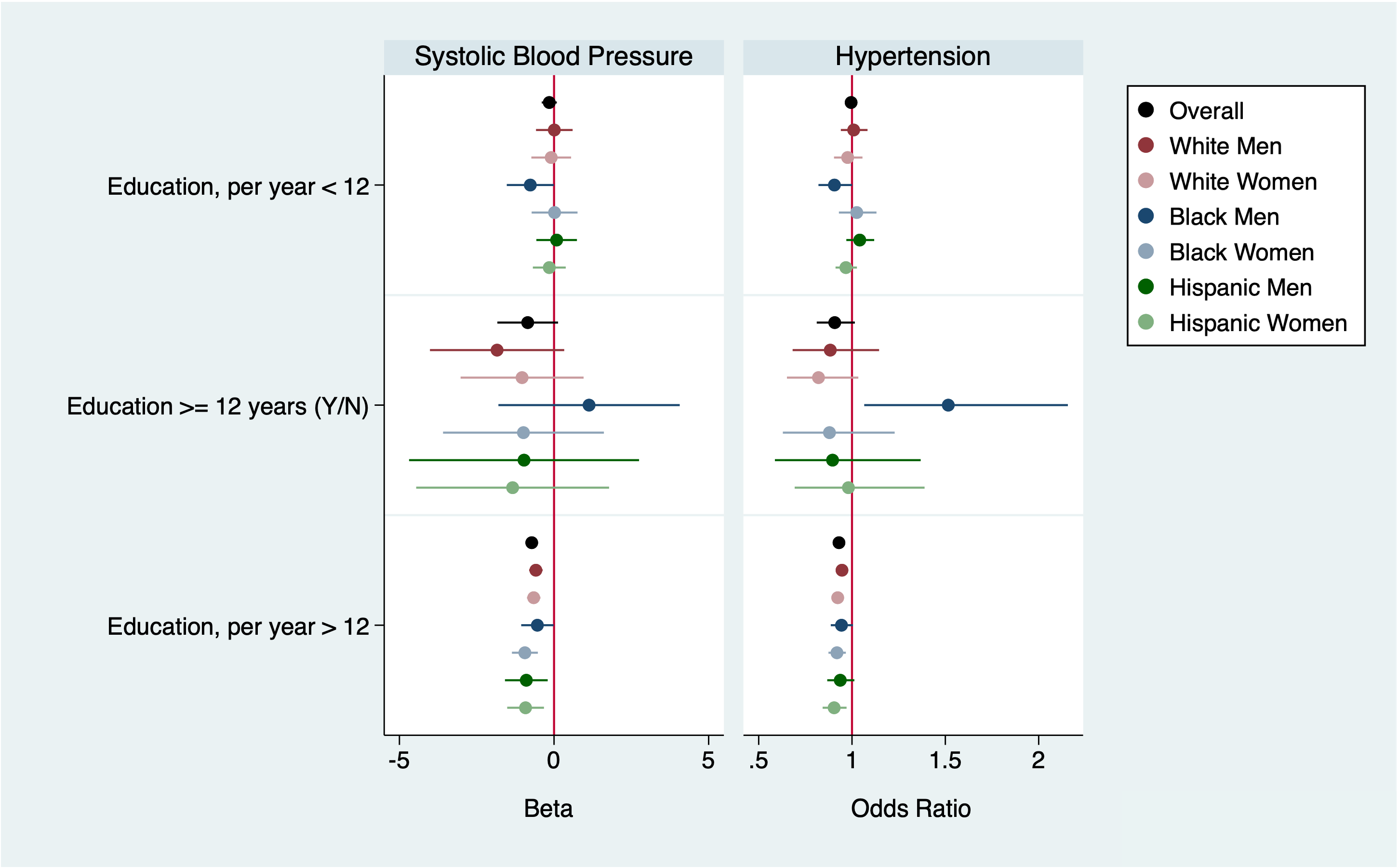


Systolic Blood Pressure was imputed using multiple imputations by chained equations with 30 iterations. Regressions are adjusted for birth year, birthplace, mother’s and father’s education, missing indicators for mother’s and father’s education, birthplace and wave of outcome measurement. Coefficient plots indicate beta coefficients for systolic blood pressure and odds ratios for hypertension.

**Table S9 - Generalized Estimating Equation, base models excluding immigrants**

|  | Systolic Blood Pressure | | | | | | | | | | | Hypertension | | | | | | | |
| --- | --- | --- | --- | --- | --- | --- | --- | --- | --- | --- | --- | --- | --- | --- | --- | --- | --- | --- | --- |
| VARIABLES | | | | β | 95% CI | | P-value | | | Odds Ratio | | | | 95% CI | P-value | |  | | |
|  | | |  | | |  | | |  | | | |  |  | |  | | |  |
| Education < 12 years | | | 0.08 | | | -0.25, 0.42 | | | 0.62 | | | | 1.00 | 0.96, 1.04 | | 0.96 | | |  |
| Education >=12 years (Y/N) | | | -1.46* | | | -2.61, -0.30 | | | 0.01 | | | | 0.89 | 0.78, 1.02 | | 0.10 | | |  |
| Education > 12 years | | | -0.76*** | | | -0.90, -0.63 | | | 0.00 | | | | 0.92*** | 0.91, 0.94 | | 0.00 | | |  |
| Race and Ethnicity (Ref=White) | | |  | | |  | | |  | | | |  |  | |  | | |  |
| Black | | | 5.56*** | | | 4.89, 6.24 | | | 0.00 | | | | 2.37*** | 2.18, 2.57 | | 0.00 | | |  |
| Hispanic | | | 2.20*** | | | 1.14, 3.26 | | | 0.00 | | | | 1.15* | 1.02, 1.30 | | 0.02 | | |  |
| Gender (Ref=Male) | | | 1.90* | | | 0.39, 3.41 | | | 0.01 | | | | 1.11 | 0.94, 1.32 | | 0.21 | | |  |
| Female | | | -4.52*** | | | -4.98, -4.07 | | | 0.00 | | | | 0.84*** | 0.79, 0.89 | | 0.00 | | |  |
| Father's Education | | | -0.09* | | | -0.17, -0.01 | | | 0.03 | | | | 0.98*** | 0.97, 0.99 | | 0.00 | | |  |
| Mother's Education | | | 0.04 | | | -0.06, 0.14 | | | 0.41 | | | | 1.00 | 0.98, 1.01 | | 0.39 | | |  |
| Missing Mother's Education | | | 0.26 | | | -0.71, 1.24 | | | 0.60 | | | | 0.93 | 0.83, 1.04 | | 0.19 | | |  |
| Missing Father's Education | | | 0.05 | | | -0.70, 0.80 | | | 0.90 | | | | 1.11* | 1.01, 1.21 | | 0.03 | | |  |
| Birthplace (Ref=Non-Southern US) | | |  | | |  | | |  | | | |  |  | |  | | |  |
| Southern birth | | | 0.94*** | | | 0.43, 1.45 | | | 0.00 | | | | 1.17*** | 1.10, 1.24 | | 0.00 | | |  |
| US not specified | | | 0.31 | | | -1.17, 1.80 | | | 0.68 | | | | 1.12 | 0.93, 1.33 | | 0.23 | | |  |
| Age | | | 0.30*** | | | 0.28, 0.33 | | | 0.00 | | | | 1.04*** | 1.04, 1.05 | | 0.00 | | |  |
| Year (Ref=2006) | | |  | | |  | | |  | | | |  |  | |  | | |  |
| 2008 | | | 0.12 | | | -0.56, 0.80 | | | 0.73 | | | | 1.01 | 0.95, 1.09 | | 0.69 | | |  |
| 2010 | | | 0.42 | | | -0.12, 0.96 | | | 0.13 | | | | 1.12*** | 1.06, 1.17 | | 0.00 | | |  |
| 2012 | | | -1.79*** | | | -2.45, -1.13 | | | 0.00 | | | | 1.04 | 0.97, 1.11 | | 0.27 | | |  |
| 2014 | | | -2.34*** | | | -2.92, -1.75 | | | 0.00 | | | | 1.04 | 0.98, 1.10 | | 0.16 | | |  |
| 2016 | | | -2.81*** | | | -3.47, -2.15 | | | 0.00 | | | | 0.98 | 0.91, 1.05 | | 0.54 | | |  |
| 2018 | | | -3.80*** | | | -4.44, -3.17 | | | 0.00 | | | | 0.98 | 0.92, 1.04 | | 0.52 | | |  |
| Constant | | | 114.53*** | | | 112.22, 116.84 | | | 0.00 | | | | 0.15*** | 0.12, 0.20 | | 0.00 | | |  |
|  | | |  | | |  | | |  | | | |  |  | |  | | |  |
| Participants (Observations) | | 21,076 (44,908) | | | | | |  | | | 21,076 (44,908) | | | | | | |  | |

Compared to the analytic sample used for the main analysis, we excluded 3,450 non-US born participants based on self-reported birth place. Missing outcomes, exposure, and covariates (except for mother’s and father’s education) were imputed using multiple imputations by chained equations with 30 iterations. Education is operationalized as a spline using self-reported years of schooling (5-17). Statistical significance is indicated as following: *** P-value <0.001, ** P-value <0.01, * P-value <0.05.

**Table S10 - Generalized Estimating Equation, interaction models excluding immigrants**

|  | Systolic Blood Pressure | | | Hypertension | | |
| --- | --- | --- | --- | --- | --- | --- |
| VARIABLES | β | 95% CI | P-value | Odds Ratio | 95% CI | P-value |
| White women | -4.47** | -7.49, -1.44 | 0.00 | 0.94 | 0.66 - 1.35 | 0.75 |
| Black men | 3.25 | -0.35, 6.84 | 0.08 | 1.18 | 0.77 - 1.80 | 0.45 |
| Black women | 2.02 | -1.38, 5.42 | 0.24 | 2.42*** | 1.59 - 3.70 | 0.00 |
| Hispanic men | 5.64 | -0.62 - 11.90 | 0.08 | 1.73 | 0.88 - 3.41 | 0.11 |
| Hispanic women | -0.16 | -5.45 - 5.12 | 0.95 | 1.05 | 0.60 - 1.83 | 0.87 |
|  |  |  |  |  |  |  |
| Education < 12 years | 0.24 | -0.39 - 0.87 | 0.46 | 1.01 | 0.93 - 1.09 | 0.84 |
| White women*education, per year < 12 | -0.43 | -1.41 - 0.56 | 0.40 | 0.97 | 0.86 - 1.09 | 0.60 |
| Black men*education, per year < 12 | -0.29 | -1.29 - 0.72 | 0.58 | 0.93 | 0.82 - 1.05 | 0.26 |
| Black women*education, per year < 12 | -0.14 | -1.14 - 0.85 | 0.78 | 1.02 | 0.89 - 1.17 | 0.76 |
| Hispanic men*education, per year < 12 | 0.55 | -0.97 - 2.07 | 0.48 | 1.10 | 0.93 - 1.30 | 0.25 |
| Hispanic women*education, per year < 12 | 0.06 | -1.28 - 1.39 | 0.93 | 1.01 | 0.88 - 1.16 | 0.90 |
|  |  |  |  |  |  |  |
| Knot (12 years) | -1.61 | -3.88 - 0.66 | 0.16 | 0.91 | 0.70 - 1.19 | 0.50 |
| White women*knot (12 years) | 0.28 | -2.86 - 3.42 | 0.86 | 0.87 | 0.60 - 1.26 | 0.46 |
| Black men*knot (12 years) | 2.19 | -1.68 - 6.06 | 0.27 | 1.69* | 1.07 - 2.68 | 0.03 |
| Black women*knot (12 years) | -0.05 | -3.69 - 3.59 | 0.98 | 0.98 | 0.63 - 1.55 | 0.95 |
| Hispanic men*knot (12 years) | -1.61 | -8.31 - 5.09 | 0.64 | 0.80 | 0.38 - 1.68 | 0.56 |
| Hispanic women*knot (12 years) | -1.67 | -7.40 - 4.06 | 0.57 | 0.91 | 0.49 - 1.66 | 0.75 |
|  |  |  |  |  |  |  |
| Education, per year > 12 | -0.56*** | -0.77 - -0.35 | 0.00 | 0.94*** | 0.91 - 0.96 | 0.00 |
| White women*education, per year > 12 | -0.32* | -0.61 - -0.04 | 0.03 | 0.97* | 0.93 - 1.00 | 0.05 |
| Black men*education, per year > 12 | 0.12 | -0.49 - 0.72 | 0.70 | 1.03 | 0.96 - 1.11 | 0.45 |
| Black women*education, per year > 12 | -0.50* | -1.00 - -0.01 | 0.05 | 0.96 | 0.90 - 1.02 | 0.16 |
| Hispanic men*education, per year > 12 | -1.08* | -2.04 - -0.11 | 0.03 | 0.91 | 0.81 - 1.02 | 0.12 |
| Hispanic women*education, per year > 12 | -1.10* | -2.03 - -0.17 | 0.02 | 0.97 | 0.87 - 1.08 | 0.55 |
|  |  |  |  |  |  |  |
| Participants (Observations) | 21,076 (44,908) | | | 21,076 (44,909) | | |

Compared to the analytic sample used for the main analysis, we excluded 3,450 non-US born participants based on self-reported birth place. Regressions are adjusted for birth year, birthplace, mother’s and father’s education, missing indicators for mother’s and father’s education, birthplace and wave of outcome measurement. Education is operationalized as a spline using self-reported years of schooling (5-17). Statistical significance is indicated as following: *** P-value <0.001, ** P-value <0.01, * P-value <0.05.

**Figure S7 – Stratified results excluding immigrants**

**
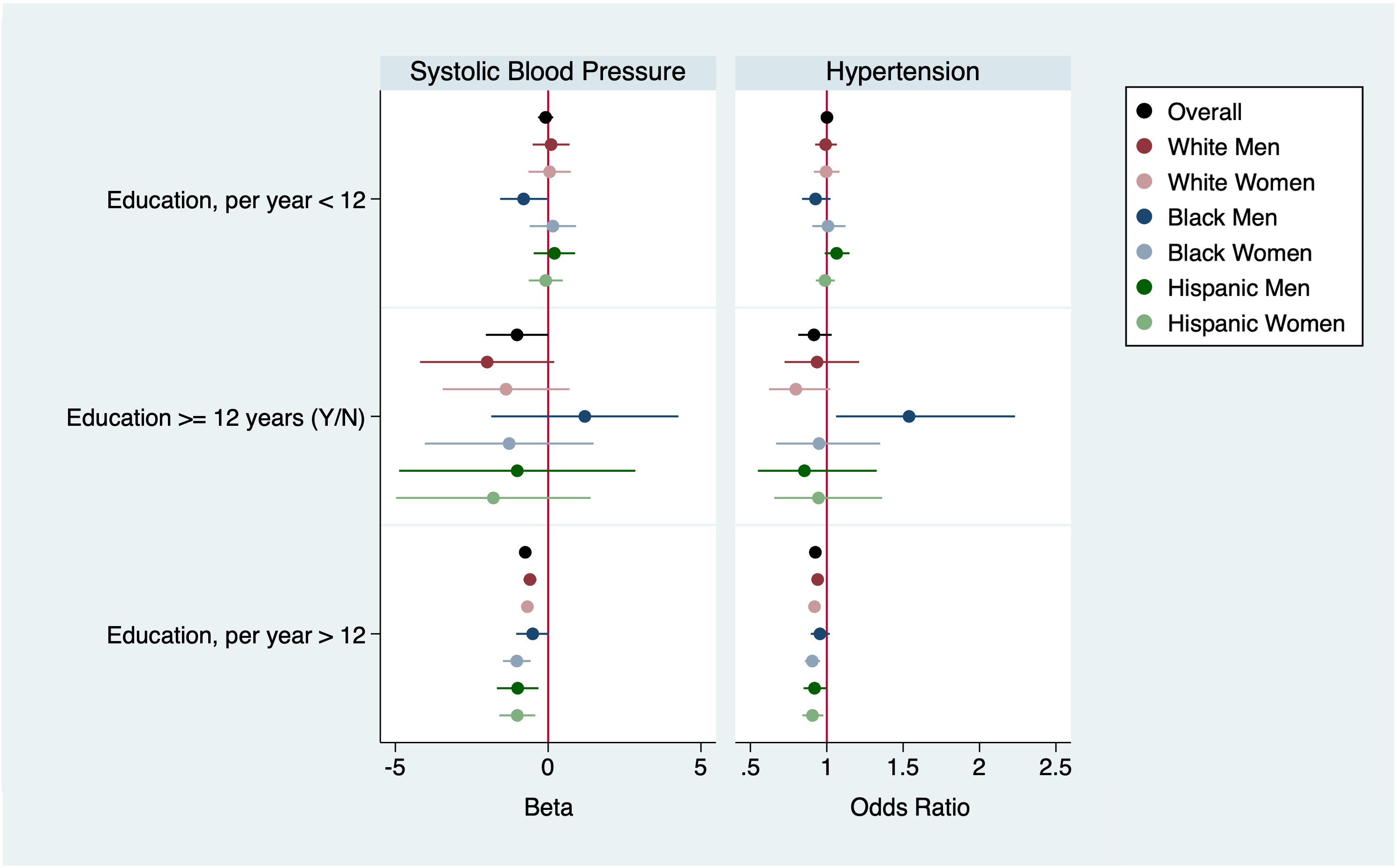
**

Compared to the analytic sample used for the main analysis, we excluded 3,450 non-US born participants based on self-reported birth place. Regressions are adjusted for birth year, birthplace, mother’s and father’s education, missing indicators for mother’s and father’s education, birthplace and wave of outcome measurement. Coefficient plots indicate beta coefficients for systolic blood pressure and odds ratios for hypertension.
